# Host plants and insecticides shape the evolution of genetic and clonal diversity in a major aphid crop pest

**DOI:** 10.1111/eva.13417

**Published:** 2022-07-08

**Authors:** Lise Roy, Benoit Barrès, Cécile Capderrey, Frédérique Mahéo, Annie Micoud, Maurice Hullé, Jean‐Christophe Simon

**Affiliations:** ^1^ Université de Lyon, Anses, INRAE, USC CASPER Lyon France; ^2^ CEFE, University of Montpellier, CNRS, EPHE, IRD Univ Paul Valéry Montpellier 3 Montpellier France; ^3^ INRAE, UMR IGEPP Rennes France

**Keywords:** agricultural landscape, gene flow, insecticide resistance, *Myzus persicae*, temporal dynamics

## Abstract

Understanding the spatiotemporal dynamics of pesticide resistance at the landscape scale is essential to anticipate the evolution and spread of new resistance phenotypes. In crop mosaics, host plant specialization in pest populations is likely to dampen the spread of pesticide resistance between different crops even in mobile pests such as aphids. Here, we assessed the contribution of host‐based genetic differentiation to the dynamics of resistance alleles in *Myzus persicae*, a major aphid pest which displays several insecticide resistance mechanisms. We obtained a representative sample of aphids from a crop mosaic through a suction trap for 7 years and from various crops as a reference collection. We genotyped these aphids at 14 microsatellite markers and four insecticide‐resistant loci, analyzed the genetic structure, and assigned host‐based genetic groups from field‐collected aphids. Four well‐defined genetic clusters were found in aerial samples, three of which with strong association with host‐plants. The fourth group was exclusive to aerial samples and highly divergent from the others, suggesting mixture with a closely related taxon of *M*. *persicae* associated with unsampled plants. We found a sharp differentiation between individuals from peach and herbaceous plants. Individuals from herbaceous hosts were separated into two genetic clusters, one more strongly associated with tobacco. The 4‐loci resistance genotypes showed a strong association with the four genetic clusters, indicative of barriers to the spread of resistance. However, we found a small number of clones with resistant alleles on multiple host‐plant species, which may spread insecticide resistance between crops. The 7‐year survey revealed a rapid turn‐over of aphid genotypes as well as the emergence, frequency increase and persistence of clones with resistance to several families of insecticides. This study highlights the importance of considering landscape‐scale population structure to identify the risk of emergence and spread of insecticide resistance for a particular crop.

## 1 INTRODUCTION

The selection pressure exerted by the application of pesticides in agriculture is an important driver of the evolution and genetic differentiation of pest populations in agricultural landscapes. Since the marketing authorizations of pesticides used for crop protection are specific to each agricultural sector and can change rapidly over time, understanding the spatiotemporal dynamics of pest populations in landscapes composed of crop mosaics is a major challenge for the sustainable management of control methods (Petit et al., 2020; Scheiner & Martin, 2020). The dynamics of resistance alleles depends on involved pesticide resistance mechanisms, their genetic basis (number of genes, level of dominance, and pleiotropic effects), and fitness trade‐offs across environments (Bourguet et al., 2000; Comins, 1977; Hawkins et al., 2019; Lenormand et al., 2018). Movement between crops of resistant individuals further influences the spread of resistance alleles and is affected by the spatial distribution of host plants, the history of insecticide authorizations, and the timing of insecticide treatments (REX Consortium, 2013). It also depends on the extent of gene flow within a pest species between different crops in agricultural landscapes, which is determined by a variety of factors, among which the intrinsic mobility of pest populations, the degree of host‐plant specialization, and the level of reproductive isolation are most important (Gauffre et al., 2015; Vasseur et al., 2013). Assessing the relative contribution of these factors to the spread of resistance alleles thus has strong implications for pest management.

Aphids are economically important pests of many crops that are heavily controlled by insecticides. As a consequence, many aphid species have evolved resistance mechanisms against most insecticides, so that managing the few efficient molecules is a challenging issue (Bass et al., 2014). Aphids generally exhibit high mobility because they regularly produce winged forms that may transit via air flows over areas of tens of kilometers (Llewellyn et al., 2004). Thus, it is assumed that aphids have no migration limit to transfer insecticide resistance alleles at a landscape scale, other than through the availability of their host plant: aphids are generally considered as highly plant‐specialized, so that different species typically infest distinct crops of the mosaics (Peccoud et al., 2009).

Plant specialization may gradually lead to reproductive isolation between host races and eventually to the formation of new species, as predicted by the ecological speciation theory (Rundle & Nosil, 2005). Thus, variation in host plant specificity within pest species can lead to differences in the spread of insecticide resistance alleles within and between crops. Within the few polyphagous aphid species (i.e., able to feed on plant species of different families), it is common to find some plant‐based genetic differentiation between populations, resulting in the existence of host races or biotypes specialized on a limited number of host plants within the species’ food spectrum (Drès & Mallet, 2002; Ferrari et al., 2006; Peccoud et al., 2010; Raymond et al., 2001; Vanlerberghe‐Masutti & Chavigny, 1998). Furthermore, polyphagous herbivores are inherently selected for the ability to mount a broad response to diverse plant defense chemistry, particularly through the production of detoxification enzymes (Bass et al., 2013; Dermauw et al., 2013). This coincidental pleiotropic effect can then be co‐opted as a resistance mechanism, with the activity of such enzymes further enhanced under insecticidal pressure by selection within pre‐existing or de novo produced variation (Hawkins et al., 2019). Finally, aphids have a particular reproductive mode, which is cyclical parthenogenesis and involves the succession of parthenogenetic (clonal) generations and a sexual one (holocyclic life‐cycle) per year. However, it is frequent to find within the same species aphid lineages having lost the sexual phase (thus reproducing continually by parthenogenesis; anholocyclic life‐cycle) co‐existing with those having sex once in a year (Simon et al., 2002). This loss of sex creates de facto a reproductive isolation between the two types of lineages, which in turn may affect the circulation of insecticide resistance genes among populations.

Here, we wanted to assess the contribution of host‐based genetic differentiation and reproductive isolation to the dynamics of insecticide resistance alleles in *Myzus persicae* (Sulzer, 1776), an economically important aphid known to have rapidly evolved resistance to all commonly used synthetic insecticides (Bass et al., 2014). Reputedly a generalist, *M*.* persicae* is capable of developing on approximately 50 cultivated hosts (Hullé et al., 2020). However, a subspecies specific to tobacco, *M*.* persicae nicotianae*, has been described (as a species, then a subspecies; Blackman, 1987; Eastop & Blackman, 2005), but its taxonomic status has been questioned (Clements et al., 2000; Clements & Wiegmann, 2000). Furthermore, populations of *M*.* persicae* with contrasting modes of reproduction (holocyclic and anholocyclic populations) coexist especially in temperate regions (Blackman, 1974). The primary host used for sexual reproduction by holocyclic populations once in a year in the fall is peaches or other *Prunus* trees and the secondary hosts used by all populations for parthenogenetic reproduction in spring and summer are many herbaceous plants.

A recent analysis of complete genomes of 127 clones of *M*.* persicae* s.l. sampled from peach trees and different herbaceous plant species throughout the world detected a sharp differentiation between clones from peach and other plants, consistent with a long history of divergence between holocyclic and anholocyclic lineages (Singh et al., 2021). Singh et al. (2021) also showed that almost all their clones from tobacco form a distinct lineage, consistent with the existence of a tobacco‐adapted race. However, some clones of *M*.* persicae* can switch from one host‐plant species to another unrelated one without substantial fitness differences (Mathers et al., 2017; Vorburger et al., 2003). If this occurs *in natura*, some more generalist clones may exploit multiple plants and spread resistance in landscape mosaics hosting various crops. The sampling design by Singh et al. (2021) allowed a valuable characterization of the global diversity of mutations affecting genes involved in insecticide resistance and provided clues regarding host‐genotype associations. However, the relatively small number of individuals studied and the unbalanced origin of the aphid host plants sampled (with for example an over‐representation of clones from tobacco) limits the general significance of these results in terms of population genetics. To fully decipher the evolutionary history of the loci of interest in relation to host specificity and to question the potential for transfer from one host to another, it is necessary to study the dynamics of allelic frequencies at the spatial scale of aphid population functioning and on different host plants in sympatry.

Eight mechanisms of resistance to the six main families of insecticides used since the first half of the 20^th^ century to control *M*.* persicae* (organophosphates, cyclodienes, carbamates, pyrethroids, neonicotinoids, and ketoenols) have been described to date (Bass et al., 2014; Singh et al., 2021). These mechanisms rely on polygenic overexpression of detoxifying enzymes (E4/FE4 esterases, organophosphate and carbamate resistance; P450 CYP6CY3 and CYP6CY4, neonicotinoid resistance), reduced penetration through the cuticle (neonicotinoid resistance), or a monogenic change in the insecticide target (acetylcholinesterase: S431F mutation, carbamate resistance; voltage‐gated sodium channel (VGSC): L1014F, M918T, M918L, M918I mutations, pyrethroid resistance; GABA‐gated chloride channel: A302G mutation, cyclodiene resistance; nicotinic acetylcholine receptor: R81T mutation, neonicotinoid resistance; acetyl‐CoA carboxylase: A2226V mutation, ketoenol resistance; Bass et al., 2014; Singh et al., 2021). Several independent mutations have been shown to cause single amino acid substitutions at position 918 of VGSC (M918L and M918I encoded by two different codons each; Singh et al., 2021). While pyrethroid resistance is generally recessive in other insects, Panini et al. (2021) showed that it is made dominant in *M*.* persicae* by the insertion of a transposable element into the susceptible allele. For each of the five oldest insecticide families, the time lag between the first approval for use and the identification of control‐compromising resistances worldwide has been estimated at 20–25 years (Bass et al., 2014). Furthermore, the relatively rapid emergence of neonicotinoid resistance resulted from a succession of selection events progressively building a genetic structure with high‐level resistance, recruiting first on pre‐existing variations, then on rarer, *de novo* variations associated with higher‐level resistances (Troczka et al., 2021). This likely results from detoxication mechanisms co‐opted by exposure to nicotine, a secondary metabolite partly similar to neonicotinoids and produced by tobacco, prior to selection by the insecticide itself, highlighting the strong interrelationship between host specialization and insecticide resistance (Troczka et al., 2021).

The objective of our study was to determine how inter‐crop migration and reproductive mode variation contribute to the dynamics of insecticide‐resistant alleles in populations of *M*.* persicae* in agricultural landscape mosaics. We chose to study how these two factors influence the temporal dynamics of resistance alleles in the Rhône Valley over a period surrounding the onset of treatment failures on oilseed rape (2001–2007). Indeed, recurrent reports of field treatment failure against *M*.* persicae* by a mixture of a carbamate and a pyrethroid have been recorded from 2005 onwards by the plant protection services on oilseed rape in the region encompassing the Rhône Valley (CETIOM, personal communication). The Rhône Valley is among temperate zones where *M*.* persicae* can be found as both holocyclic and anholocyclic populations (Blackman, 1974).

In order to ensure the representativeness of the intra‐population diversity and to work on an area consistent with the scale of population functioning of the species, we took advantage of sampling by a 12‐m high aerial suction trap surrounded by a mosaic of diverse crops in the Rhône Valley. As the aphid individuals in the air sample are part of the aerial plankton of an area surrounding a suction trap by a few tens of kilometers (Llewellyn et al., 2004), they constitute a random sample of the diversity of genotypes in the surrounding landscape. Because the landscape surrounding the aerial trap was a mosaic of crops including three crops of interest (oilseed rape, tobacco, and peach), the sample was representative of *M*. *persicae* populations developing on oilseed rape, tobacco, peaches, and other plants in sympatry. In order to potentially infer the host plant of origin of the aphid individuals sampled following this minimized bias protocol, we constituted a reference collection of *M*. *persicae* samples collected in several locations in France, in different years, and on identified host plant including the crops targeted by our study.

To assess the extent of genetic differentiation due to plant specialization and reproductive mode variation, we characterized the microsatellite multilocus genotypes (MLG) and multilocus resistance genotypes (resistotypes) of both the air‐trapped aphids and the reference collection of aphids sampled from identified crops. We found substantial genetic differentiation strongly associated with differences in resistotypes, indicating the existence of barriers to the spread of insecticide resistance alleles. However, we also found a small number of generalist clones with resistant alleles that persist over time and may contribute to the circulation of insecticide resistance between crops.

## 2 MATERIALS AND METHODS

### 2.1 Biological material

Two sets of individuals were included in our study: the first set was obtained using an aerial suction trap in order to monitor temporal changes in genetic diversity (including insecticide target site resistance) while minimizing sampling bias. The second set was a targeted sampling of different identified plant species. The latter constitutes reference samples of *M*.* persicae* in France from specifically identified crops, dedicated to infer the plant origin of the aphids of the aerial sample (first set) and thus to assess the influence of the host plant on the genetic structure of the air‐sampled aphid population.

The first set of samples (referred to as the “aerial sample” hereafter) consisted of winged individuals of *M*.* persicae* caught using a 12.2 m high suction trap being part of a permanent network for monitoring aphid migrations in France and in operation since 1974 (i.e., Agraphid French suction trap network, Hullé et al., 1987). The trap was located close to Saint‐Marcel‐lès‐Valence (Auvergne Rhône‐Alpes region, France; trap position: 44.977381°, 4.930401°), in the Rhône Valley. The Rhône Valley is a highly diversified agricultural region, with irrigated meadows, vineyards, various vegetable, fruit, and herbaceous crops alternating with considerable wooded areas (Chambre d’Agriculture Auvergne‐Rhône‐Alpes, 2021). Aphids used in the present study were collected between 2001 and 2007. The trap was surrounded by a heterogeneous landscape, dominated by a variety of crops, including peach trees, the primary host of *M*. *persicae*, and a diversity of secondary hosts such as oilseed rape, potato, tomato, eggplant, sweet pepper, sunflower, and tobacco. Aphids were collected from the suction trap daily and preserved in 95% ethanol. Of these, 100 individuals per year were randomly selected and subjected to individual genotyping.

The second set of individuals (referred to as the “host plant population sample” in the rest of the manuscript) consisted of wingless females sampled from 17 different plant hosts (mainly oilseed rape, tobacco, and peach tree; Zenodo repository; see Data Availability): 604 aphid females were sampled from 37 oilseed rape plots spread across northeastern France between 2009 and 2013 (mostly same samples as in Roy et al., 2013), 62 from tobacco plots located in more than 38 different localities across France between 2003 and 2008, 102 from three peach orchards in 2011–2013 and 76 from other miscellaneous herbaceous crops in the Rhône Valley in 2014 (eggplant, chard, cabbage, zucchini, bean, orange tree, pepper, potato, lettuce, soybean, tomato, tamarillo, sunflower, verbena). They were integrated into the present study as a reference to infer the host plant origin of aerial suction trapped samples based on individual clustering analyses. In order to represent the diversity at the field/orchard level, leaves from several dozen plants/trees bearing aphids were sampled from fields/orchards and separated from each other during transfer to the laboratory. There, a single female per plant/tree was randomly selected and stored in independent 2 ml microtubes filled with 95% ethanol until DNA extraction.

### 2.2 DNA extraction

DNA extraction from individual aphids was performed using NucleoSpin^®^ 96 Tissue kit (Macherey‐Nagel), following the manufacturer recommendations with one modification: the sample lysis step consisted of grinding each aphid in 180 µl of T1 buffer with 1 stainless steel ball of 4.76 mm diameter at 20 Hz for 30 s twice. The DNA was eluted with a two‐step procedure using each 30 µl of BE buffer preheated to 70°C for a final volume of 60 µl of DNA solution.

### 2.3 Microsatellite development and genotyping

A new set of microsatellite markers was developed for *M*.* persicae* using a high‐throughput method based on coupling multiplex microsatellite enrichment and next‐generation sequencing on 454 GS‐FLX Titanium platforms (Malausa et al., 2011). The QDD pipeline (Meglécz et al., 2010) was used to analyse the 454 sequences and design primers for amplification of the detected microsatellite motifs. A set of 48 microsatellite loci was first selected from 521 validated by QDD based on the following criteria: perfect motifs, high number of repeats, dinucleotide motifs, Tm comprised between 59°C and 61°C, GC percentage between 45 and 55, and length of PCR products. This set was thereafter used on a subsample of *M*.* persicae* populations using the following protocol.

We used the M13‐tailed primer method (Boutin‐Ganache et al., 2001) to label amplicons for visualization on the capillary sequencer. Forward primers were 5′‐tailed with a 23‐bp M13 sequence. Loci were amplified in a final volume of 10 µl polymerase chain reaction (PCR). The reaction mixture contained 2 µl of total DNA, 0.35 µM of each primer, 0.8 mM of a four‐nucleotide mixture, 2 mM of MgCl_2_, 2 µl of PCR Buffer (Promega), and 0.4 U of Taq DNA Polymerase (Promega). The M13 primers were 5′‐fluorescently tagged with PET, 6‐FAM, NED, or VIC at 0.35 µM for assessment of allele sizes on a capillary sequencer (see below). PCR was conducted on S1000 thermal cycler (2008, Bio‐Rad Laboratories) using the following cycling conditions: initial denaturation at 95°C for 5 min, first cycle of DNA amplification (repeated 20 times) with a denaturation step at 95°C for 1 min, hybridization at 60°C for 1 min, elongation at 72°C for 90 s; the second cycle of M13 amplification with 20 repetitions of the following steps: 95°C for 1 min, 53°C for 1 min, and 72°C for 90 s. The PCR ended with a final elongation at 72°C for 10 min. Diluted PCR products (1.2 μl on 2.4 μl water) were added to 10 μl of Hi‐Di formamide (Applied Biosystems) containing 0.8% of 500 LIZ DNA ladder (Applied Biosystems), and electrophoresis was performed in the capillary sequencer ABI 3730 (Applied Biosystems). Allele calls were automatically assigned by GENEMAPPER (version 3.7, Applera Corp) and visually checked. Fourteen loci showing a high polymorphism rate and being easy to score have been eventually retained (Table 1).

**TABLE 1:** Sequence information and general characteristics of the 14 microsatellite markers designed for this study on *Myzus persicae*

Locus	Number of alleles	Allele range	% null homozygote	Ho	Primer forward	Primer reverse	Genomic position
MP_2	8	[329–362]	0.15	0.21	ATTCCCGTCTGAACGATACG	GTCCCACCAGAAACCCATAA	scaffold_6:28679865‐28680221
MP_4	7	[288–321]	0.00	0.3	ATATAGCTGGGTGGGATGGA	CGAGTTTTCACGACAGAACG	scaffold_4:51945413‐51945721
MP_5	6	[341–350]	0.38	0.6	TTTTAAAGGGCACTCGGG	AGGAATAAACGGGGGCAT	scaffold_5:22673642‐22673989
MP_7	7	[180–199]	0.08	0.42	TACAGTGTTTGTCCGTTGCC	ACGTAAGCTTTTCGTCCTGC	scaffold_6:16830385‐16830404
MP_9	17	[180–230]	0.23	0.63	TCTACGGCGAATAAGTGGCT	TTAGTAGCCTTACCTCCAACGC	scaffold_3:55056009‐55056205
MP_13	5	[245–256]	1.69	0.28	GTTGTATACCCCTGGAGATTCG	CTCTAAAGAAGTTCCTGCAGCC	scaffold_3:69699916‐69700166
MP_23	16	[350–408]	7.16	0.67	GACTGCAGTGCGTTTTATGC	ATGTGGGTAGGTGCCGTTAT	scaffold_1:92900558‐92900912
MP_27	9	[238–273]	1.15	0.14	CTGACAACCCCTCAAAAAGC	AAACCTCCATGGACAACGAC	scaffold_2:5930284‐5930527
MP_28	4	[346–361]	0.23	0.55	ATATTAAGACGGTGGTGCCG	TATAGACGCGCACAGATTCG	scaffold_3:46587990‐46588344
MP_38	32	[160–243]	0.31	0.88	AGTCGTGGTTACGCAATCCT	TCGGTAAACGAGCATCAGTG	scaffold_2:81941716‐81941903
MP_39	15	[361–403]	0.08	0.75	ACACAGTGCCTACACAGCGTAT	TGATGTTTCCATGGACCG	scaffold_1:63042446‐63042834
MP_44	10	[162–200]	1.69	0.53	GTTCGTGTTTGTTGACCCCT	TGTGAAACGTCGAAACCG	scaffold_1:43462272‐43462446
MP_45	10	[309–332]	0.15	0.58	CAACCTCATATCGCTCACGA	AGACGACGGTCGACGATAAT	scaffold_1:52205573‐52205897
MP_46	11	[322–361]	0.38	0.84	ACGACGTGTGTGCATAAAGC	TGGACCGACCATCATCACTA	scaffold_1:40667667‐40668010

Note

Ho, observed heterozygosity. Genomic positions are given from the version assembled in Mathers et al. (2017). Scaffold numbers refer to the different chromosomes of *M*. *persicae* (2*n* = 12).

We performed three multiplexed PCRs with fluorescently labeled primers on 1446 individuals. Loci were amplified in a final volume of 10 µl polymerase chain reaction (PCR). The reaction mixture contained 2 µl of total DNA, between 0.25 and 0.5 µM of each primer, 0.8 mM of a four‐nucleotide mixture, 2 mM of MgCl_2_, 2 µl of PCR Buffer (Promega), and 0.4 U of Taq DNA Polymerase (Promega). PCR was conducted on S1000 thermal cycler (2008, Bio‐Rad Laboratories) using the following cycling conditions: initial denaturation at 95°C for 5 min, a cycle of DNA amplification repeated 30 times with a denaturation step at 95°C for 1 min, hybridization at 54°C for 1 min, elongation at 72°C for 1 min. The PCR ended with a final elongation at 72°C for 10 min. Diluted PCR products (1.2 μl on 2.4 μl water) were added to 10 μl of Hi‐Di formamide (Applied Biosystems) containing 0.8% of 500 LIZ DNA ladder (Applied Biosystems), and electrophoresis was performed in the capillary sequencer ABI 3730 (Applied Biosystems). The alleles were automatically called by GENEMAPPER (version 3.7, Applera Corp) according to the ladder. The chromatograms of both the ladder and the SSR fragments electrophoresis were visually checked to eliminate any wrong assignation due to automatic analysis by the software.

Individuals with two or more missing microsatellite information were not considered in subsequent analyses. The combination of the information of the different loci constitutes the neutral multilocus genotype (MLG).

### 2.4 Target site resistance genotyping

Individuals were genotyped for four loci on three genes associated with target site resistance against three commonly used insecticide families: carbamates, pyrethroids, and neonicotinoids. The genotype on position 431 of the *ace* gene coding acetylcholinesterase 2 (AChE2, 2 alleles: wild 431S and mutant 431F, named MACE) was assessed by derived cleaved amplified polymorphic sequence (dCAPs) PCR. The 25 µl final volume of the reaction mixture contained 0.6 µM of each primer (Mace_dCaps_sens 5′‐TAGTAACTCCGAAGAGGGTTACGATT‐3′ and Mace_dCaps_reverse 5′‐CTGGGTCATTTGGGCTGAAC‐3′), 12.5 µl of 2× Qiagen Multiplex PCR master mix (Qiagen), and 1 µl of total DNA. The PCR cycles consisted of an activation step with the Taq polymerase enzyme at 95°C for 15 min, followed by 30 cycles of a denaturation step at 95°C for 60 s, a hybridization step at 54°C for 60 s, and an extension step at 72°C for 30 s. A final extension step was performed at 72°C for 5 min. After amplification, 10 µl of the PCR product were digested in a final volume of 20 µl with 1 µl of 10 U/µl *Hinf*I restriction enzyme (New England Biolabs) and 2 µl of CutSmart 10× buffer (New England Biolabs) at 37°C for 6 h. The resulting restriction profiles were evaluated using the QIAxcel^®^ system with a screening cartridge and the default method “AM420.” The sizing of the fragments was enabled by the addition of QX Size Marker 100 bp‐2.5 kb and QX Alignment Marker 15 bp–3 kb. The genotypes at positions 918 and 1014 of the *para* gene coding the voltage‐gated sodium channel (VGSC) were assessed at once by Sanger sequencing (PCR conditions as in Cassanelli et al., 2006 and sequencing as in Fontaine et al., 2011). For the few individuals with more than one double peak in the codon at position 918 (on chromatogram), sequencing of individual alleles was performed by cloning as in Roy et al. (2013). Position 918 is the super‐*kdr* position, with 4 alleles known at the beginning of the study: wild 918M (codon = ATG), standard *skdr* 918T (codon = ACG), atypical *skdr* 918L associated with two different codons: TTG (Fontaine et al., 2011) and CTG (Panini et al., 2014). We will refer to these two alleles as follows: L‐ttg and L‐ctg. Position 1014 is the *kdr* one, with 2 alleles known to date (the wild 1014L and mutant 1014F). Finally, the R81T mutation linked to resistance against neonicotinoids was detected using the dCAPS PCR described in Mottet et al. (2016). Note that the insecticide resistance loci and microsatellite loci analysed in this study are not linked neither physically because their respective positions on the *M*. *persicae* genome are at least 7.2 Mb apart (Table 1) nor due to population genetics factors (see below).

### 2.5 Genetic data analyses

#### 2.5.1 Microsatellite quality assessment

Linkage disequilibrium between all microsatellite marker pairs was computed using “genepop” R package (Rousset, 2008) with the Markov chain parameters set to default on clone‐corrected (i.e., only one copy of each MLG) datasets for the host populations and for the genetic clusters (see below). Linkage disequilibrium analysis between all markers (microsatellite and target site resistance markers) was also conducted on the individuals sampled from peach trees. The general characteristics of the new markers (number of alleles, allele range, percentage of null allele) were evaluated on the complete combined datasets.

#### 2.5.2 MLG assignment

We used “Rclone” (Bailleul et al., 2016) to both assign the MLG of every aphid with complete data and assign the MLG of individuals with one missing data by inferring it from the closest complete MLG. A minimum spanning network based on the matrix of pairwise genetic dissimilarity distances between MLG was built using the “poppr” v2.9.2 R package (Kamvar et al., 2014) for the aerial trap sampling individuals. Genetic cluster membership or resistance genotype information for the *kdr*, *skdr*, and MACE mutations was represented using color codes for the network nodes. The R81T mutation information was not displayed since this mutation emerged only a few years after the aerial trap sampling and was therefore not detected in this set of samples.

#### 2.5.3 Genetic cluster analyses

An individual‐centered Bayesian clustering approach was used to describe the genetic structure of the samples using STRUCTURE v2.3.4 (Falush et al., 2003). The analysis was performed on the combined sets of sampling (aerial trap and host population individuals). Because this analysis relies on minimizing the linkage disequilibrium within groups, only one copy of each MLG was kept to avoid artifact from clone repetition. One hundred independent runs were conducted for K ranging from 1 to 15 using the admixture model with correlated allele frequencies. Each run consisted of a burn‐in period of 100,000 iterations followed by 1,000,000 simulations. The K values of interest were assessed using the maximization of Pr(X|K), the Delta‐K method (Evanno et al., 2005) and by identifying for each K the major modes among the 100 runs using CLUMPAK (Kopelman et al., 2015; Supporting information). The final Q‐matrix for each K of interest was obtained by averaging Q‐matrices of the runs belonging to the major mode. Individuals were assigned to a genetic cluster with a threshold of *q* > 0.7. Because the underlying assumption of the model‐based approach may be violated due to the biological characteristic of the studied species (especially its capacity for asexual reproduction), the results of the Bayesian clustering approach were supplemented by a discriminant analysis of principal components (DAPC), a multivariate nonparametric method, using “adegenet” R package (Jombart et al., 2010).

To estimate the level of differentiation at the population level between genetic clusters, pairwise genetic *F*_ST_ were calculated (estimated and averaged over loci, Weir & Cockerham, 1984), and population genotypic differentiation was tested for significance using exact tests (Goudet et al., 1996) using “genepop” R package (Rousset, 2008).

#### 2.5.4 Genetic diversity

Genotypic and genic diversity were assessed for each host plant population and for each genetic cluster from the aerial trap sampling using R. The microsatellite genotypic diversity was characterized by calculating G:N (ratio of the number of identified MLG to the number of individuals sampled) and the Pielou's evenness of the MLG (Arnaud‐Haond et al., 2007; equation 16). Genic diversity was described by estimating allelic and private allelic richness (A_r_ and PrA_r_, respectively) using the rarefaction method (El Mousadik & Petit, 1996), and Nei heterozygosity (H_e_). The association between the resistance genotypes (resistant homozygote, resistant heterozygote, and sensitive homozygote) and host plants of origin (peach, oilseed rape, and tobacco) was tested using the Fisher's exact test for count data on our field samples for the four target site resistance genotypes (*kdr*, *skdr*, MACE, and R81T). We also tested the association between the resistance genotypes and genetic clusters on the aerial sample for only three target site resistance genotypes (*kdr*, *skdr*, and MACE), since R81T was absent from these data. The analyses were performed on contingency tables using the R function “fisher.test.”

### 2.6 Phylogenetic analysis of the barcode regions of the CO1 coding gene

In order to complete our understanding of the delineated genetic groups and to check for conspecificity of the genotyped individuals, we sequenced the barcode region of cytochrome oxidase 1 from 24 individuals of the aerial sample (taken at random) using primers LepF1 (5′‐ATTCAACCAATCATAAAGATATTGG‐3′) and LepR1 (5′‐TAAACTTCTGGATGTCCAAAAAATC‐3′) and PCR conditions described in Hebert et al. (2004). The absence of stop codons was verified with Seaview v5.0.4 (Gouy et al., 2010). The obtained sequences were subjected to a nucleotide‐nucleotide megaBLAST using the Basic Local Alignment Search Tool (BLAST) to see whether specimens referenced in Genbank and assigned to other species were more closely related to the individuals with the most divergent sequences in our sample than to *M*.* persicae*. We also searched the sequences of different *Myzus* species in the Barcoding of Life (BOLD) database (Ratnasingham & Hebert, 2007). We grouped the first sequences retained by BLAST (the most similar to ours), those of the different *Myzus* species and our new sequences in a fasta file, and aligned them using the ClustalOmega algorithm in Seaview. The phylogenetic analysis of the alignment was performed using the Maximum Likelihood (ML) method with PhyML (Guindon et al., 2010). The model was selected with the SMS tool (Lefort et al., 2017) following the BIC criterion. Pairwise identity percentages were calculated with ClustalX v2.1 (Larkin et al., 2007; Thompson, 1997).

### 2.7 Complementary datasets

In order to approximate the relative proportions of target crops and the temporal evolution of their area in the suction trap region, we collected the yearly surfaces of crop hosts under scrutiny in the present study in the Rhône‐Alpes region from the Agreste's database (Statistique Agricole Annuelle, https://agreste.agriculture.gouv.fr/agreste‐web/). In addition, to identify any obvious link between the history of insecticide use and the dynamics of resistotypes and resistant alleles, we used data on Marketing Authorizations (MA) and the first release in France for insecticide molecules used on the three main crops under study (peach tree, tobacco, oilseed rape) from 2001 to 2007 documented in the Index Acta Phytosanitaire series and the French government website e‐phy (http://e‐phy.agriculture.gouv.fr/).

## 3 RESULTS

### 3.1 New set of *Myzus persicae* microsatellite markers

Out of 1446 individuals, 1299 were genotyped successfully (i.e., with a maximum of one missing information for the 14 microsatellites). Neither evidence for scoring error due to stuttering nor evidence for large allele dropout were detected with any loci. The mean number of alleles per marker identified on the 1299 genotyped individuals was 11.2 (ranging from 4 to 32, Table 1). The mean percentage of null homozygotes evaluated on the complete dataset was 0.98% (ranging from 0.00% to 7.16%, Table 1).

### 3.2 Temporal variation in abundance of aphids caught at the suction trap

Due to failure in amplifying some or all of the 14 microsatellite markers, we recovered genotypic data from an average number of 65 ± 11 aphids/year (out of 100). Most of these failures were probably due to a lower quality of the biological material leading to poor DNA quality: a higher proportion of failures was recorded in the oldest samples.

### 3.3 Genetic structure according to host (MLG + RG)

In field samples, clonal diversity estimated from G:N and Pielou's evenness was substantially higher on herbaceous hosts than on the primary host, as expected (Table 2). Allelic and private allelic richness were highest in “Other crops,” consistent with host‐related differentiation in this multi‐host sample (Table 2). The very small G:N, low Pielou equitability, and low allelic richness values on oilseed rape were consistent with populations largely dominated by a very small number of clones on this crop (Roy et al., 2013; Zamoum et al., 2005; Table 2). Significant linkage disequilibrium between microsatellite markers, evaluated on the clone‐corrected dataset, was only found rarely when considering the peach tree population (2 out of 91 LD tests) while they were frequent for the populations sampled on herbaceous hosts (50/91, 9/78, and 41/91 for oilseed rape, tobacco, and other crops, respectively). No significant linkage disequilibrium was found between microsatellite and target site resistance markers in the peach tree population (Figure S1) and very few in the red cluster (Figure S2).

**TABLE 2:** Genotypic and genic diversity indices by host populations

Crops	G:N	J′	A_r_	PrA_r_	H_e_
Peach	0.68 (69/102)	0.96	4.95	0.51	0.46
Oilseed rape	0.06 (39/604)	0.43	3.62	0.28	0.43
Tobacco	0.23 (14/62)	0.74	4.57	0.63	0.48
Other crops	0.30 (23/76)	0.72	5.24	1.17	0.46

Abbreviations: A_r_, allelic richness; G:N, ratio between the number of genotypes and the number of individuals; H_e_, Nei heterozygosity; J′, Pielou's Evenness; PrA_r_, private allelic richness.

**FIGURE 1:**
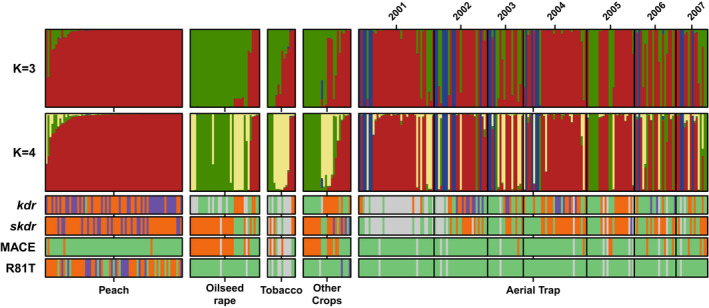
Genetic structure of *Myzus* aphids sampled in the air and on different crops. Top two lines, Q plot from the structure analysis of microsatellite multilocus genotypes, with K = 3 and K = 4. A vertical line represents an individual, and the proportion of its assignment to each cluster is represented by the colored segments. The colors of the clusters identified in K = 4 are used throughout the paper to designate the associated genetic clusters (red, green, yellow, and blue). Lower lines, visualization of resistance genotypes identified in each individual, with the following color code: light green, susceptible homozygote, purple, resistant homozygote, orange, heterozygote carrying the susceptible wild‐type allele and a mutant allele, gray, missing data. *kdr* and *skdr* loci are linked to pyrethroids target site resistance, MACE locus to pirimicarb target site resistance, and R81T locus to neonicotinoids target site resistance. The sampling sources (different crops and aerial trapping) are indicated at the bottom. The sampling years for the aerial trapping are indicated at the top

Based on Evanno's Delta‐K method (Evanno et al., 2005), the optimal number of clusters in Bayesian analyses was K = 3 when estimated on the whole clone‐corrected dataset (field and aerial samples; Figure 1; details in Figures S3 and S4). All three clusters were recorded in the aerial samples. In field samples, the red cluster was recorded mainly from peach trees and the green cluster only from secondary hosts (oilseed rape, tobacco, and other plants). The blue cluster was only found in aerial samples. With K = 4, the green 3‐K cluster is split into two groups (green and yellow), while the red and the blue clusters kept delineated in the same way as with K = 3. The divergence between the blue and the other clusters was marked, with the membership coefficient to this cluster inferred by Bayesian clustering close to 100% for individuals assigned to this blue cluster, while individuals assigned to other clusters shared a more important part of the membership coefficient with remaining clusters (Figure 1). The 4‐K yellow and green clusters were both recorded from tobacco and oilseed rape, with contrasting distributions (green cluster more frequent on oilseed rape and yellow cluster more frequent on tobacco; Table 3).

**TABLE 3:** Genetic diversity of the 4‐K clusters

Diversity indices	Red cluster	Green cluster	Yellow cluster	Blue cluster
G:N	0.71 (103/145)	0.21 (19/89)	0.15 (25/171)	0.54 (25/46)
Pielou's evenness	0.92	0.68	0.53	0.92
Allelic richness (A_r_)	4.81	2.73	4.06	3.39
Private allelic richness (PrA_r_)	1.24	0.39	0.66	2.13
Nei heterozygosity (H_e_)	0.47	0.42	0.43	0.40
Distribution of aphids per sampling origin (read by line)				
% in aerial trap	32.2	19.7	37.9	10.2
% in tobacco	9.7	37.1	53.2	0.0
% in oilseed rape	1.3	80.7	18.0	0.0
% in peach tree	100.0	0.0	0.0	0.0
% in other crops	6.9	83.3	9.7	0.0
Resistance genotypes per locus in aerial samples				
% of sensitive kdr genotypes	43.1	100.0	27.9	97.5
% of sensitive *skdr* genotypes	43.5	45.5	95.8	100.0
% of sensitive MACE genotypes	100.0	46.6	92.9	100.0
% of sensitive R81 genotypes	100.0	100.0	100.0	100.0

Note

G:N, ratio between the number of genotypes and the number of individuals.

The segregation of resistance genotypes in the different loci perfectly reflected the sharp differentiation revealed by microsatellite markers between populations from peach trees and herbaceous hosts: resistant homozygotes at the *kdr* and *skdr* loci recurrently found on peach whilst absent from herbaceous hosts, R81T present on peach, absent on herbaceous hosts and absent from the aerial sample, MACE mutation almost only on aphids from herbaceous hosts (Figure 1; *p *< 0.001 for the four Fisher's exact tests for count data in the air sample). A similar pattern was noted when considering the genetic clusters in the aerial sample (higher frequency of homozygous resistant genotypes for *kdr*, *skdr*, absence of MACE in the peach‐associated cluster; *p *< 0.001 for the three Fisher's exact tests for count data).

### 3.4 Aerial sample

**FIGURE 2:**
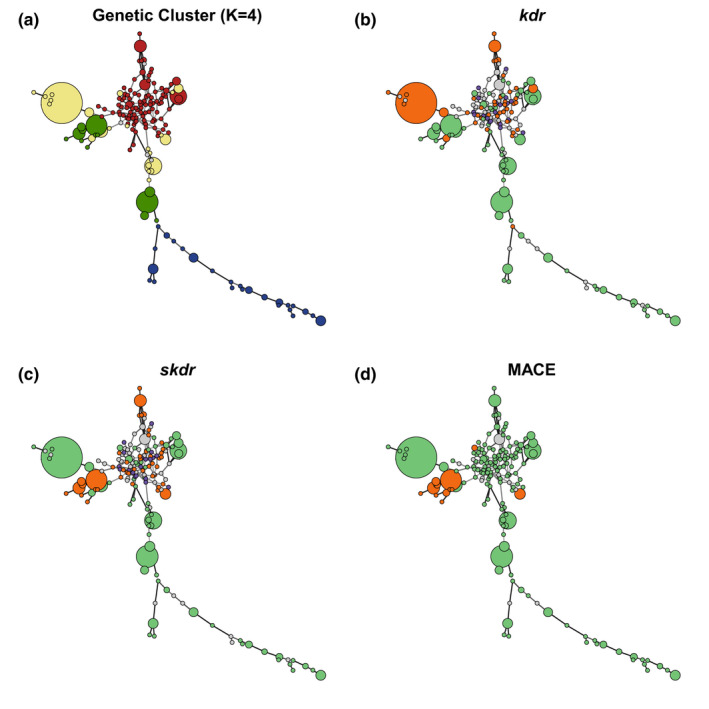
Multilocus genotypes network (microsatellite data) with genotypes colored according to their respective genetic clusters (a) and with their resistance genotypes at the three loci of interest *kdr* (b), *skdr* (c), and MACE (d). *kdr* and *skdr* loci are linked to pyrethroids target site resistance and MACE locus to pirimicarb target site resistance. Note that several resistant alleles were encountered at the *skdr* locus (b), so resistant homozygotes (purple) and heterozygotes (orange) have various genotypes

In aerial samples, the red and yellow clusters represented each ca. 1/3 of aphids, while the green and blue clusters represented ca. 1/5 and 1/10, respectively (Table 3). G:N and Pielou's evenness showed contrasts between the red cluster versus the yellow and green clusters similar to those observed between primary versus herbaceous hosts, respectively (Table 3). The private allele richness of the blue cluster was higher than the others, a further indication of the marked divergence of this population (Table 3). The blue cluster was predominantly detected during the first semester, i.e., in spring (93%), and was strongly differentiated from the three other clusters (*F*_ST_ = 0.525 with the red cluster, *F*_ST_ = 0.548 with the 3‐K green cluster, and *F*_ST_ = 0.506 with the yellow cluster). The *F*_ST_ values between the three clusters encountered on the sampled crops were much smaller (0.106–0.274; Table S1). Similar patterns were observed with DAPC clustering and in the DAS tree (see Figures S5–S7). The network of MLGs belonging to the red cluster showed an intensely reticulated structure rich in single MLGs, whereas the ones for the green, yellow and blue clusters showed less connections, with the first two incorporating many repeated MLGs (Figure 2a).

### 3.5 Persistence of MLGs over the period

**FIGURE 3:**
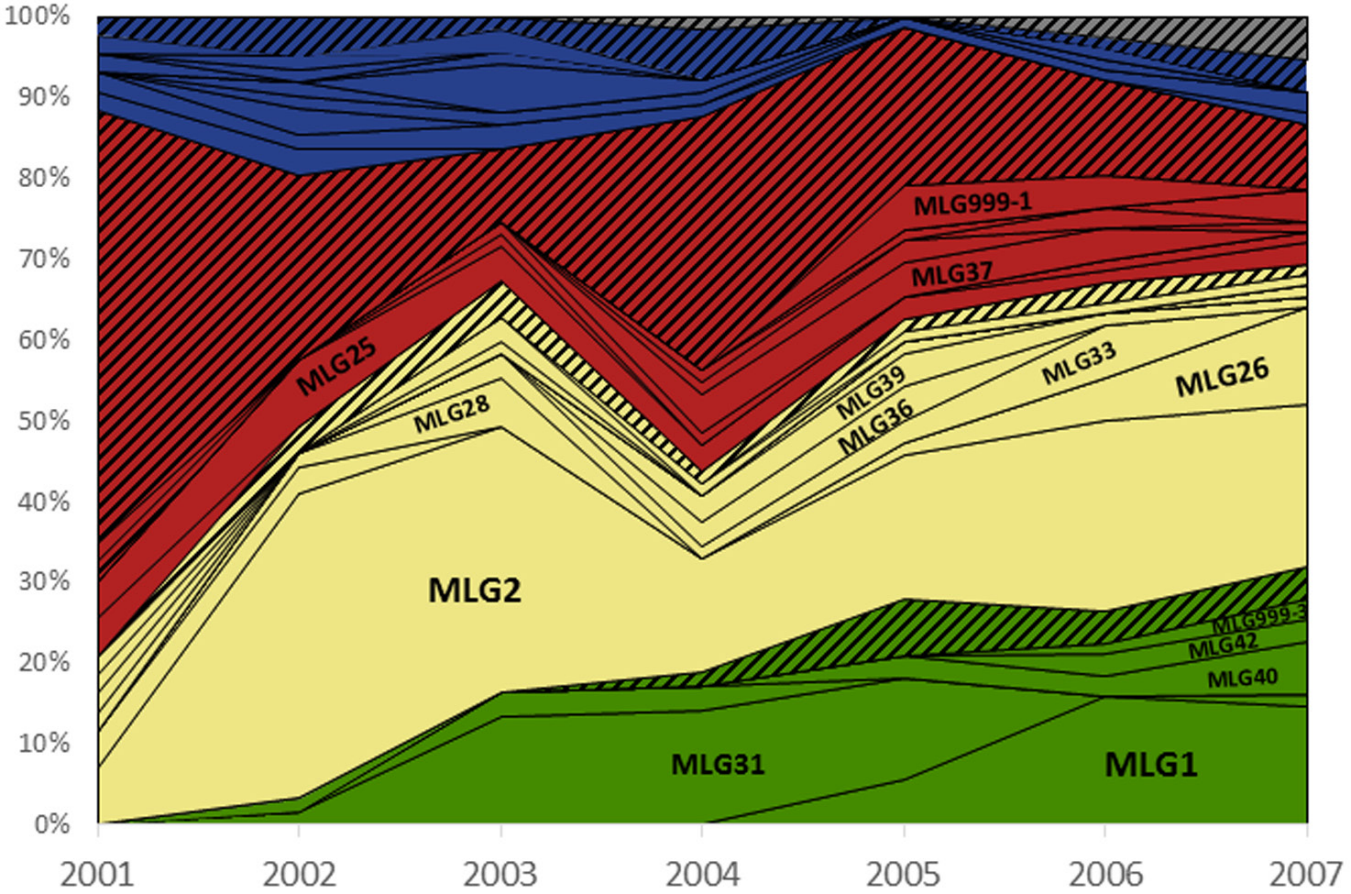
Copy number of multilocus genotypes (MLG) per year over the aerial trapping period. Colors refer to the 4‐K cluster assignment of individuals in Figure 1 (unassigned individuals in gray). Solid colors: repeated MLG; shaded colors: unique MLGs. The first recurrent treatment failures with carbamate in France were recorded in 2005

The red and yellow clusters were largely present over the entire aerial sampling period while the green cluster appeared to develop in the fields around the trap from 2002 and gradually increased until the end of the period. The blue cluster was present over the period but mainly in spring. By far, most of the recorded MLGs were unique or repeated with a few copies (2–6) over the whole period in the aerial dataset, especially in the red cluster, as expected. However, six MLGs provided ≥10 copies (MLG1, MLG2, MLG25, MLG26, MLG31, MLG33), with up to 100 copies for MLG2. Some of the repeated MLGs persisted over the whole period (e.g., MLG2, MLG25; see Figure 3), a few others disappeared (e.g., MLG36), and a few emerged and rapidly increased in frequency during the period, especially MLG1. MLG1 and clone O identified in the United Kingdom by Fenton et al. (2010) are the same clones according to microsatellite genotyping of the United Kingdom clone kindly provided by Gaynor Malloch. Interestingly, MLG25 and MLG37 were among the few multi‐copy MLGs in the red cluster and recorded almost over the entire aerial sampling period.

### 3.6 Resistant genotypes (RGs) and genetic clusters

**FIGURE 4:**
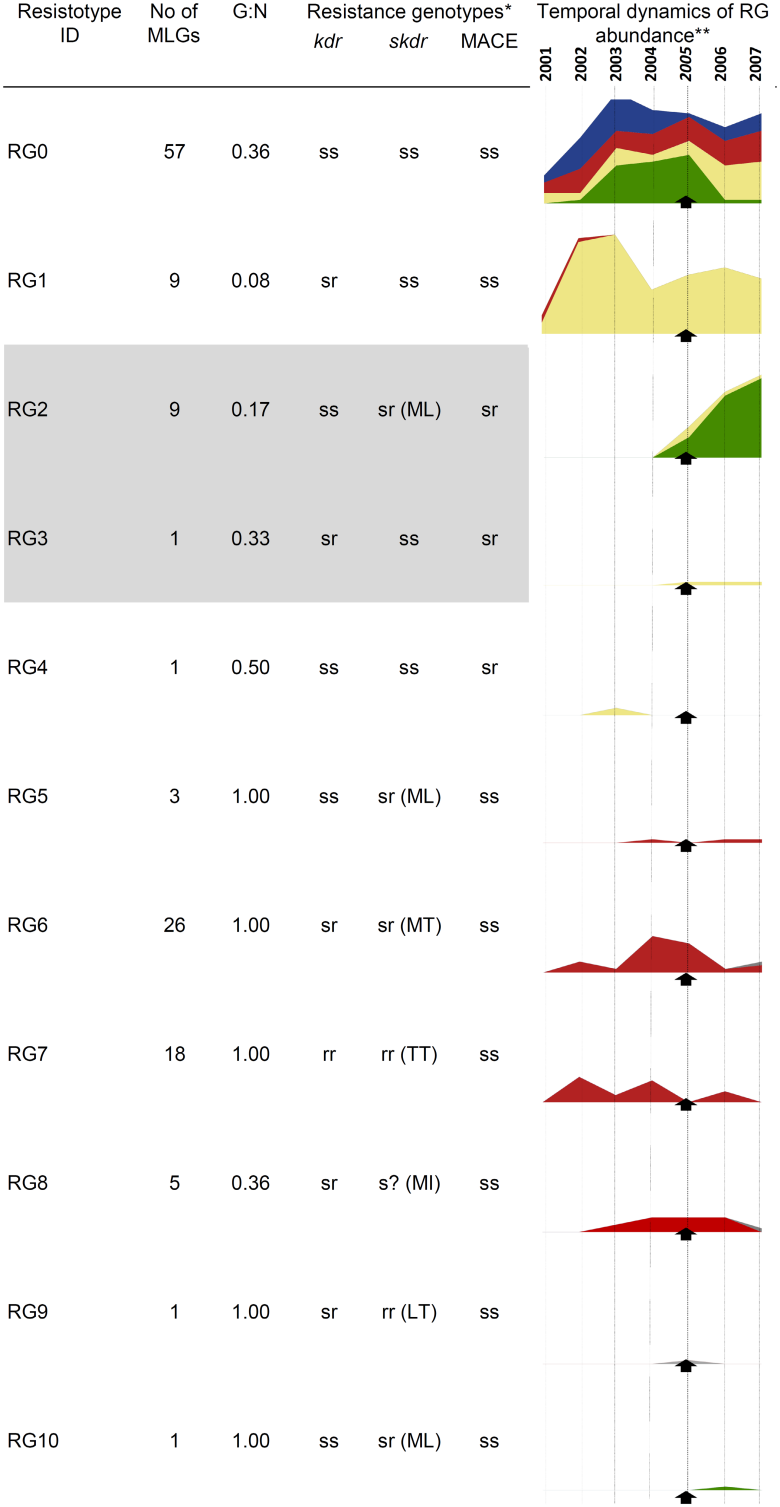
Temporal dynamics of 3‐locus resistotypes (RG) encountered in aphid individuals from the aerial sample. Individuals whose 3‐loci resistotype was not completely characterized (one or more unidentified loci; 12.7% of the aerial sample) were discarded. MLG: multilocus genotype; G:N: ratio between the number of genotypes and the number of individuals. *ss, sensitive homozygous; sr, heterozygous; rr, resistant homozygous, as known from literature in terms of associated phenotype. *kdr*: s = L, r = F; *skdr*: s = M, r = T, L, I; MACE: s = S, r = F. **The values on the vertical axis represent the number of genotyped individuals and the horizontal axis the years, shown above the first graph. Colors refer to the 4‐K cluster assignment of individuals in Figure 1 (unassigned individuals in gray). Black arrow, date of first records of recurrent failures with carbamate in oilseed rape in France

We defined resistotype as consisting of the combination of codon alleles found on the *kdr* position 1014, the *skdr* position 918, and the MACE position 431 (as in Roy et al., 2013). Note that we have also analysed alleles at position 81 of the β1 subunit of the nicotinic receptor of acetylcholin (nAChR) but only found the mutant allele 81T (associated with resistance to neonicotinoids, Bass et al., 2011) in field samples. This confirmed the later emergence of this mutation (as field samples were collected after aerial ones) and led us to consider here only the resistotypes formed by the three aforementioned resistance loci. Information on resistance genotypes found in the present study is provided in Table 3 and Figure 4. In the aerial dataset, 40% of the aphids carried the susceptible resistotype (homozygous susceptible allele at all studied loci). Among the remaining 60% of aerial aphids, two RG with two mutant targets were recorded (RG2 and RG3), with mutant alleles on the coding genes of VGSC (either *kdr* or *skdr*) and of AChE (MACE mutation; Figure 4). However, only RG2, a resistotype reported in Roy et al. (2013), was repeated in many copies.

RG2 gathers the *skdr* L‐ttg mutation on VGSC and the MACE mutation (S431F) on AChE. In the present study, the former was only found in RG2 (i.e., associated with MACE) except in a single individual caught in 2006. The MACE mutation was found alone (not associated with 918L) in only five individuals during the aerial sampling period. RG2 was found associated with nine MLGs, five of which had clonal copies. Their sequence of emergence in our dataset was as follows: 2004, detection of MLG39 (yellow cluster); 2005, detection of MLG1 and MLG40 (green cluster); 2006, detection of MLG42 (+ some unique MLGs; green cluster); 2007, detection of MLG999‐3 (+ some unique MLGs; green cluster; see Figures 2b,c and 3). Except for the genotypes in a single copy, all were recorded during the years following their first detection, but only MLG1 developed massively (Figure 3). The first detection of RG2 was associated with the yellow cluster in 2004; however, obviously, the most important cluster possessing the RG2 over the 2004–2007 period was the green cluster. Specifically, RG2 seems to have emerged from a single lineage within the green cluster as shown by the structure of the MLG network (Figure 2). The MACE mutation (S431F) was detected in 2003 and the five individuals carrying MACE, but lacking L‐ttg (RG3 or RG4) were all assigned to the yellow cluster (with q > 0.70).

Only two MLGs were associated with more than one resistotype. Their detection sequence over time was consistent with the gain of a resistant mutation on one allele: shift from RG0 (completely susceptible; four aphids in the air sample caught in 2004, 2005, 2005, 2007) to RG5 (mutation M/L‐ctg on the *skdr* locus; one aphid collected from other crops in 2014; MLG38, red cluster) or from RG10 (M/L‐ttg on the *skdr* locus; one aphid in the air sample caught in 2006) to RG2 (M/L‐ttg + MACE; two aphids in the air sample caught in 2007; MLG999‐3, green cluster).

### 3.7 Phylogenetic analysis of the genetic clusters

Twenty‐four randomly selected individuals from the different genetic clusters allowed a good quality amplification of the CO1 sequence. Sequencing of CO1 yielded two haplotypes: a first one found in individuals assigned to the red, green, and yellow clusters and another one, differing by 2%, found in individuals of the blue cluster (Figure S8). The first haplotype was assigned to *M*.* persicae*, with a percentage of 100% identity (query cover 100%; *E*‐values = 0.00). The second haplotype was clearly a sister group of *M*.* persicae* sequences on the selected phylogenetic topology. We did not find any sequence assigned to *M*.* antirrhini* (Macchiati, 1883) or *M*.* certus* (Walker, 1849; the closest species to *M*.* persicae*) in BOLD or in Genbank. Therefore, we could not check whether the second haplotype belongs to a species taxonomically close to *M*.* persicae* or to a strongly divergent population of the *M*.* persicae* s.l. group. However, we could verify that the second haplotype did not correspond to the tobacco‐specific clade highlighted by Singh et al. (2021), as it was again unambiguously positioned as an outgroup.

### 3.8 Complementary data

In the Rhône‐Alpes region, the mean area covered by oilseed rape fields was 14,629 ± 1407 ha until 2004, then increased each year and reached 24,237 in 2007 (Table 4). The proportion of oilseed rape area was very high compared with the other two main crops under scrutiny (on average 6 times the peach area and 23 times the tobacco area). However, we could not estimate what areas were covered by the other plants colonized by *M*. *persicae* (cultivated and noncultivated). The trap was located in an area of the Rhone Valley close to a particularly rich tobacco‐growing area. The crop protection products authorized for use on the target crops during the aerial sampling period are presented in Table 5.

**TABLE 4:** Area of crops targeted during the aerial sampling period (in Ha)

Year	Oilseed rape	Tobacco	Peach
2001	16,254	837	4340
2002	14,400	820	3937
2003	14,996	858	3537
2004	12,864	789	3033
2005	17,038	778	2619
2006	21,483	716	2285
2007	24,237	649	2027

**TABLE 5:** Authorized insecticides per year (chemical family level)

Insecticides	2000	2001	2002	2003	2004	2005	2006	2007
Organophosphates (OP)	OP	OP	OP	OP	OP	OP	OP	OP
Carbamates	OPT	OPT	OPT	OPT	OPT	OPT	OPT	OPT
Pyrethroids	OPT	OPT	OPT	OPT	OPT	OPT	OPT	OPT
Neonicotinoids	P	P	P	P	P	P	PT	PT
Organochlorines (OC)	P	P	P	P	P	P	P	P
Pyridine	P	P	P	P	P	P	P	P
Rotenon	P	P	P	P	P	P	P	P
Pyrethroids + OP	OP	OP	OP	OP	OP	OP	OP	OP
Pyrethroids + carbamates	OPT	OPT	OPT	OPT	OPT	OPT	OPT	OPT
OC + OP	O	O	O	O				

Abbreviations: O, oilseed rape; P, peach; T, tobacco.

## 4 DISCUSSION

The genetic analysis of *Myzus* aphids caught in a suction trap, over a period during which insecticide treatment failure was observed, allowed for effective monitoring of populations and resistotypes present in a mosaic of crops. Our aerial sample captured genotypes present in different crops and allowed us to record fluctuations over time with some clone replacements. The dramatic increase in the RG2 resistotype concomitant with reports of treatment failure reinforces the usefulness of such monitoring to understand the emergence and spread of new insecticide resistance mechanisms and to evaluate their impact on specific crops.

Given the dispersal ability of *M*.* persicae*, aphid individuals caught in the suction trap were randomly sampled from the aerial plankton so that their genetic composition is representative of the diversity of genotypes and resistotypes in the surrounding landscape. The presence of a separate genetic group (blue cluster in our study) of *Myzus* aphids, absent from crop samples, indicates that it develops either on wild host plants or on some specific crops excluded from the study. From the analysis of the CO1 barcode region, this genetic group appears to be a specific entity on its own, distinct from *M*. *persicae*, and from other species of *Myzus* likely to be present in the region concerned and for which CO1 sequences are available in international libraries. The complete sequence identity between this group and *M*.* polaris* suggests a close relationship with the latter species, which is, however, recorded exclusively in cold regions (Greenland, Iceland, Canada; Richards, 1963). It is possible that the genetic group recorded in the aerial trap corresponds to *M*. *certus*, which occurs in temperate zones in Europe on Caryophyllaceae and Violaceae and can be confused with *M*.* persicae* (Hullé et al., 2020). As we had no reference sample of *M*.* certus* for genotyping and comparison with our data, we cannot confirm the species identity of the divergent genetic group in our aerial sample.

### 4.1 Barriers to gene flow between *Myzus* samples on peach trees and on herbaceous hosts

In line with Hlaoui et al. (2019) and Singh et al. (2021), a strong differentiation between *Myzus* aphids sampled on peach trees and those from herbaceous plants was observed at both microsatellite markers and insecticide resistance loci. Rather than reflecting a host‐based differentiation due to the specialization of some *M*.* persicae* populations on peach, we think this genetic divergence is due to differences in the reproductive mode. Indeed, peach is the primary host of *M*.* persicae* on which sexual reproduction occurs once in a year. Hence, we expect to find from this host aphid populations undergoing regular recombination, generating large genotypic diversity. In contrast, samples collected from secondary, herbaceous, hosts are more likely to include an important proportion of aphid genotypes that reproduce asexually year‐round, and would then be genetically isolated from those found on peach trees. Consistent with this hypothesis are the high genotypic and allelic diversities, the rarity of loci in linkage disequilibrium, and the strong reticulation of the MLG network in the peach‐associated group (red cluster in our study). This is in sharp contrast with the low number of MLGs, the strong linkage disequilibrium between loci, and the presence of branches and star patterns around highly repeated MLGs in the other clusters associated with secondary hosts, suggestive of essentially asexual reproduction for quite some time. Although conducted over a larger geographic area than the aerial sampling and over a different time period, the reference sampling on identified hosts allowed unambiguous association between identified genetic clusters and populations sampled on specific host plants. One cluster gathered individuals sampled on peach, the primary host of *M*. *persicae*, and two clusters gathered individuals from herbaceous hosts (oilseed rape and tobacco). The concordance between the results of previous studies and ours strongly suggests that host‐based differentiation is maintained over time and space. Interestingly, at least two clonal lines (MLG25 and MLG37), which belonged to the peach‐associated cluster, were repeatedly found over several years, which suggests they may have lost sexual reproduction capacity more recently. The large difference in frequency of resistotypes between the peach‐associated cluster and the other secondary host‐associated clusters in the aerial sample further supports a gene flow reduction due to isolation between sexually and asexually reproducing genotypes that strongly affect the dynamics of insecticide resistance alleles in *M*.* persicae* populations.

### 4.2 Barriers to gene flow due to specialization in secondary hosts

The analysis of *Myzus* samples from the secondary hosts considered in this study showed a weak but significant differentiation between populations from tobacco and oilseed rape. In the other herbaceous crops, no specific genetic cluster was detected suggesting these crops are exploited by similar *Myzus* genotypes found either on tobacco or oilseed rape. The detection of a genetic cluster with some association with tobacco (yellow cluster) is consistent with earlier studies reporting the existence of a tobacco‐adapted race in *M*.* persicae* and equipped with specific mechanisms to overcome the toxicity of nicotine expressed in large quantities by this host plant (Bass et al., 2013; Singh et al., 2021). However, we did not find a strict association with tobacco in the genetic profiles of *Myzus* found on this plant. This result contrasts with the recent work of Singh et al. (2021), which revealed a single tobacco lineage including the vast majority of worldwide clones collected from this host in their study and characterized by more than 1 million SNPs. Our data derived from a much larger sample representative of local population diversity, although based on a reduced genome overview, clearly indicate that the tobacco‐associated cluster detected in our study was not restricted to tobacco as we sampled it from oilseed rape and other herbaceous crops. The ability of tobacco‐derived populations of *M*.* persicae* to develop well on other hosts in the laboratory had been reported previously (Nikolakakis et al., 2003; Semtner et al., 1998). A broadening of the host range of tobacco‐adapted populations may have been prompted locally by the very high and increasing proportion of oilseed rape fields during the period, as host expansion may induce host switching even in specialist herbivores (Trowbridge & Todd, 2001). The transient broadening of the host range of the tobacco lineage may be a step in the diversification of *M*.* persicae*, as hypothesized for other herbivores (oscillation theory; Janz & Nylin, 2008). Thus, it can be assumed that insecticide resistance phenotypes that may develop on tobacco are somewhat more likely to pass onto oilseed rape than vice versa. This hypothesis is supported at the clonal level as copies of some MLGs were found on both oilseed rape and tobacco, including clones with resistotypes bearing resistant alleles. For example, the RG2 resistotype containing the *skdr* L‐ttg mutation typical of oilseed rape (Singh et al., 2021), was present in both tobacco and oilseed rape field samples. It was associated with the two MLGs that were in the majority in oilseed rape from 2009 (MLG1 and MLG4), one of which was also very frequent in tobacco at least in 2007–2008 in France (MLG1: 32.3% aphids in our samples).

### 4.3 Temporal dynamics of resistotypes

Complementary to and in agreement with the study by Singh et al. (2021) that highlighted the diversity of amino acid substitutions and associated codons in a worldwide collection of clones of *M*. *persicae*, our study shows that similar independent mutations occur within aphid populations and provides an overview of the emergence scenario of these mutations. Among the resistotypes carrying resistant alleles in the aerial sample, only two were frequent and recurrent (RG1 and RG2), and the other eight were recorded intermittently or occasionally. Only RG2 showed a sharp increase during the period, which corresponds to treatment failures with carbamates reported since 2005. It is associated with resistance to both pyrethroids and carbamates and was the most frequent in *M*.* persicae* from oilseed rape (>80% of aphids in 2009–2011 samples from this crop).

We found many cases of double‐target resistance, combining mutations 431F and 918L (respectively, on insecticide targets AChE and VGSC), essentially represented by the RG2 resistotype in the aerial and on oilseed rape samples (Table S2). Of the two mutations RG2 carries, the 918L mutation on VGSC (i.e., L‐ttg at the *skdr* locus, Fontaine et al., 2011) was detected in our aerial sample for the first time in 2004. In contrast, the other mutation, MACE on AChE, was already present at least in 2003 in our samples and even earlier according to other studies (at least 1996 in the United Kingdom, Foster et al., 1998; 2001 in France, Zamoum et al., 2005). Interestingly, Singh et al. (2021) reported also in 2004 their first clone carrying the L‐ttg mutation (a clone collected from oilseed rape in the United Kingdom) and 93% of the clones carrying the L‐ttg mutation were carrying the full RG2 resistotype (almost all in the heterozygous state). The convergence of results from our large local population‐level data and the global data of Singh et al. (2021) on a sparser sample underlines the particular success of the RG2 resistotype. The temporal dynamics of the L‐ttg mutation at the *skdr* locus in *M*.* persicae* is consistent with the features of de novo mutations, typically associated with a high level of dominance over the wild‐type allele and therefore able to spread more rapidly than recessive standing mutations (Comins, 1977; Hawkins et al., 2019). Although it cannot be stated with certainty, this de novo mutation appears to have had a strong selective advantage in association with the older MACE mutation. This advantage may be explained by a compensation effect of the respective fitness costs of two mutant alleles as observed in mosquitoes with mutations on the same targets (VGSC and AChE) associated with resistance phenotypes to the same insecticide families (Berticat et al., 2008). The withdrawal of combinations of OCs + OPs on oilseed rape from 2004 may have increased selective pressure on both targets at the same time by favoring the use of the combination lambda‐cyhalothrin (pyrethroid) and pirimicarb (carbamate) on oilseed rape.

The success of RG2 does not seem to result from a continuous trajectory towards optimum, since similar de novo mutations appeared in different clonal genetic backgrounds and the success of clones equipped with this combination was very heterogeneous. For example, the first RG2‐carrying clone detected (MLG39) remained at a low frequency while the frequency of MLG1 (which also carries RG2) increased sharply from 2005. Interestingly, from 2006 onwards and for several years, MLG1 (= clone O) was recorded in very large numbers in the United Kingdom (Fenton et al., 2010), confirming the ecological success of this specific clone on large geographical scales. Although we cannot rule out stochastic effects, the contrasted success amongst the different RG2‐carrying clones suggests epistatic interactions with mutant alleles.

A similarly complex evolutionary trajectory of organophosphate resistance in a mosquito in the 20th century led to an equilibrium involving several mutant alleles with deleterious pleiotropic effects in the homozygous state maintained by overdominance (maximum fitness in heterozygotes; Labbé, Berthomieu, et al., 2007). This unexpected balance is explained by the fact that several mutations consisted of heterogeneous duplications (duplication on the same chromosome of the wild‐type allele and the mutant), which produces a kind of permanent heterozygosity (Labbé, Berticat, et al., 2007; Milesi et al., 2017). In our study, although no duplication was pointed out by Singh et al. (2021), the two mutant alleles responsible for the failures of pyrethroid + carbamate treatments since 2005 have been encountered almost only at the heterozygous status (= resistotype RG2) in clones assigned to the yellow and green clusters where asexual reproduction seems to predominate. On the primary host peach, double‐target resistotypes remained rare (2.94%), despite the presence of 918L in 1/3 of the individuals and a frequency of MACE similar to the one in the herbaceous‐associated cluster (Tables S2 and S3). This may reflect a short‐term selective advantage of asexual reproduction in the presence of co‐adapted genes (Panini et al., 2021): sexual recombination, by breaking the association, substantially slows or even prevents the spread of the advantageous genotype (Lynch, 1984), unless overdominance can be established (Labbé, Berthomieu, et al., 2007; Labbé, Berticat, et al., 2007; Milesi et al., 2017).

## 5 CONCLUSION

The combination of genetic data from air samples and individuals collected on different host plants allowed us to assess the influence of different barriers to gene flow on the spread of insecticide resistances between crops and to document their temporal dynamics in relation to pesticide use. Our result indicates a clear effect of reproductive mode variation in *M*.* persicae* on the development of resistance and circulation of insecticide resistance alleles between populations and crops. Although less pronounced, host‐based differentiation in *M*.* persicae* further contributes to restrict the spread of resistance alleles at a landscape scale. We highlighted the increase in the frequency of aphid clones with specific resistotype coincidental with treatment failure, but also large fluctuations in genotypes and resistotypes over a 7‐year period. Whether these fluctuations are driven by fitness costs associated with carrying different insecticide‐resistant alleles deserves further study.

## CONFLICT OF INTEREST

The authors have no conflict of interest to declare.

## Supporting information

Supplementary Material

## Data Availability

All data and R code for analysis are available from a Zenodo repository (https://doi.org/10.5281/zenodo.4553749). The CO1 DNA sequences generated from aerial samples can be found in the GenBank repository (accession numbers MZ578551–MZ578574).
