# Supplementary material for: Host plants and insecticides shape the evolution of genetic and clonal diversity in a major aphid crop pest
Source: Evol Appl. 2022 Jul 8;15(10):1653–69. doi: 10.1111/eva.13417 (PMC9624068; doi:10.1111/eva.13417)
Supplement: Supplementary file 1 — Supplementary Material [file EVA-15-1653-s001.pdf]

Supplementary materials

Figures S1 to S8………………………………………………………………………………………………… p. 1

Tables S1 to S2……………………………………………………………………………………………………… p. 15

Supplementary bibliography……………………………………………………………………………… p. 16

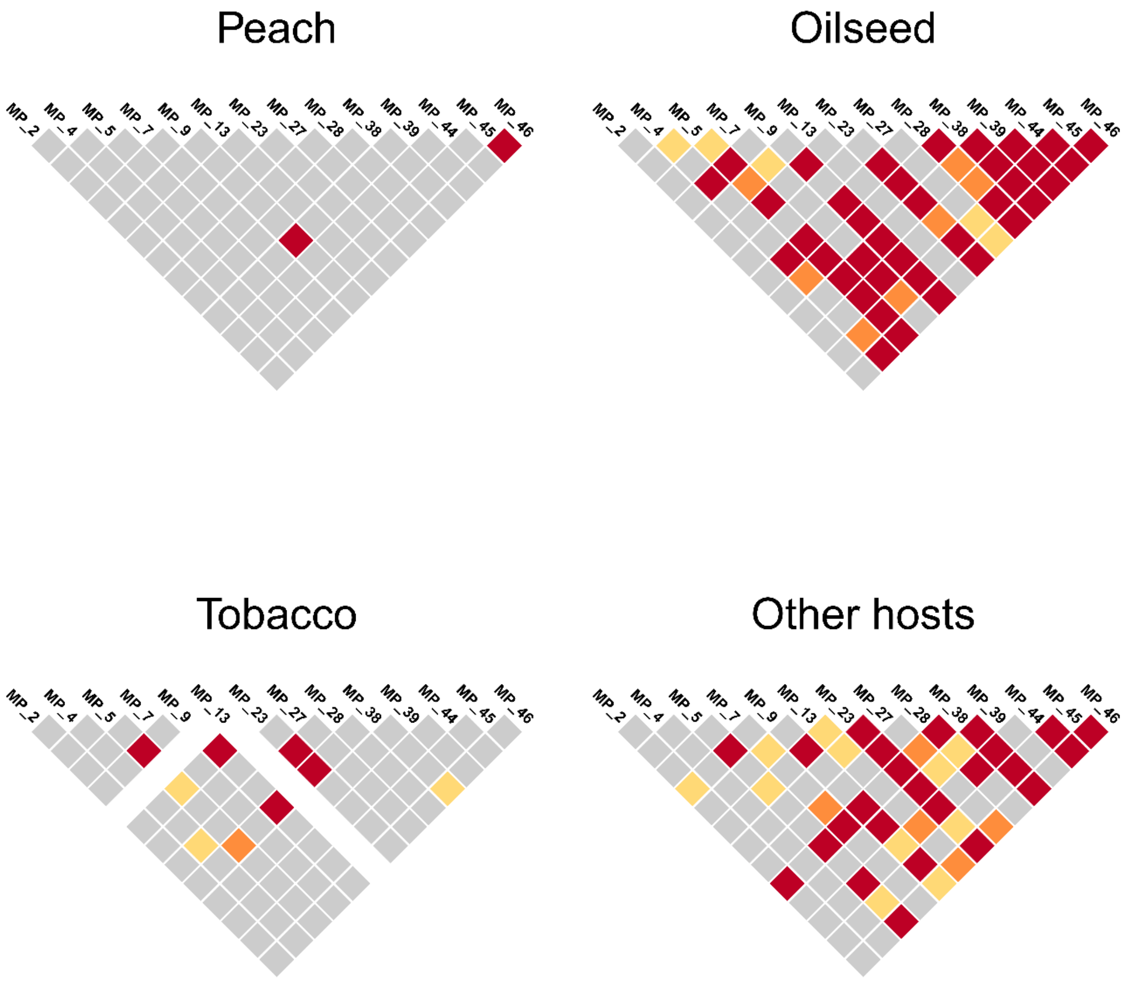

Figure S1: Linkage disequilibrium between pairs of microsatellite markers for the four host populations of *M. persicae* sampled on peach tree, oilseed rape, tobacco or other crops. Highly significant (P<0.000011), very significant (P<0.00011) and significant (P<0.00055) linkage disequilibrium are represented by red-, orange- and yellow-colored squares, respectively. Non-significant linkage disequilibria are represented by a grey-colored square. The impossibility of evaluating linkage disequilibrium between two markers in one population is indicated by the absence of a square.

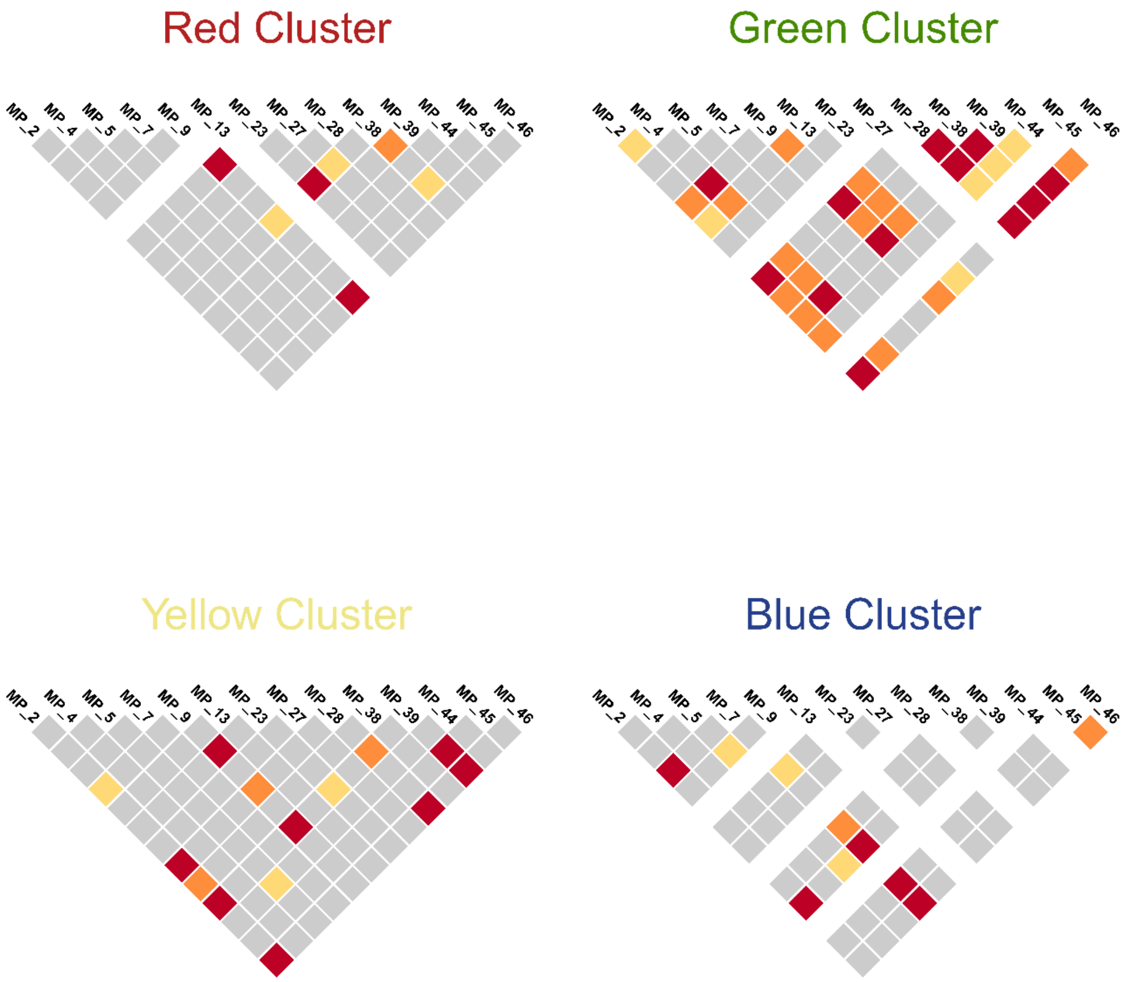

Figure S2: Linkage disequilibrium between pairs of microsatellite markers for the four genetic clusters of *M. persicae* sampled in the aerial trap. Highly significant (P<0.000011), very significant (P<0.00011) and significant (P<0.00055) linkage disequilibrium are represented by red-, orange- and yellow-colored squares, respectively. Non-significant linkage disequilibria are represented by a grey-colored square. The impossibility of evaluating linkage disequilibrium between two markers in one population is indicated by the absence of a square.

Fig. S3 and S4: Determining the best K for the Bayesian clustering analyses

After 100 STRUCTURE runs for each K on the clone-corrected dataset, the Q-matrices obtained were analyzed through the main pipeline of CLUMPAK (Kopelman et al 2015). The CLUMPP ‘LargeKGreedy’ algorithm with 2000 repeats was used with a threshold for similarity scores of

0.80 and a threshold for minimum cluster size of 5 (*i.e.,* 5% of 100 runs). For K=2 to 6, we then plotted the 100 runs ordered according to the mode(s) obtained (Fig. S3) with individuals grouped by host on which they were sampled from. In order to identify the K of interest, we also computed the ad-hoc statistic Delta K (Fig. S4).

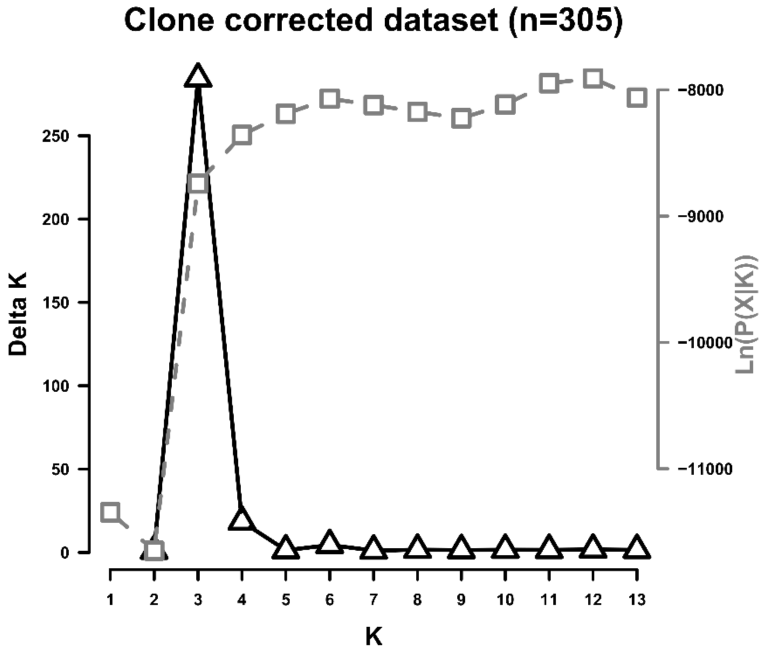

Figure S3: Plot of the variation of the Ln(P(X|K) and the Delta-K.

The two values of K of interest based on the Delta K and the CLUMPAK analyses were 3 and 4. The major modes include 100 and 76 runs with a mean similarity score between runs of 0.996 and 0.996 for K values of 3 and 4, respectively. The final Q-matrices were obtained by averaging the Q-matrices runs of the major modes.

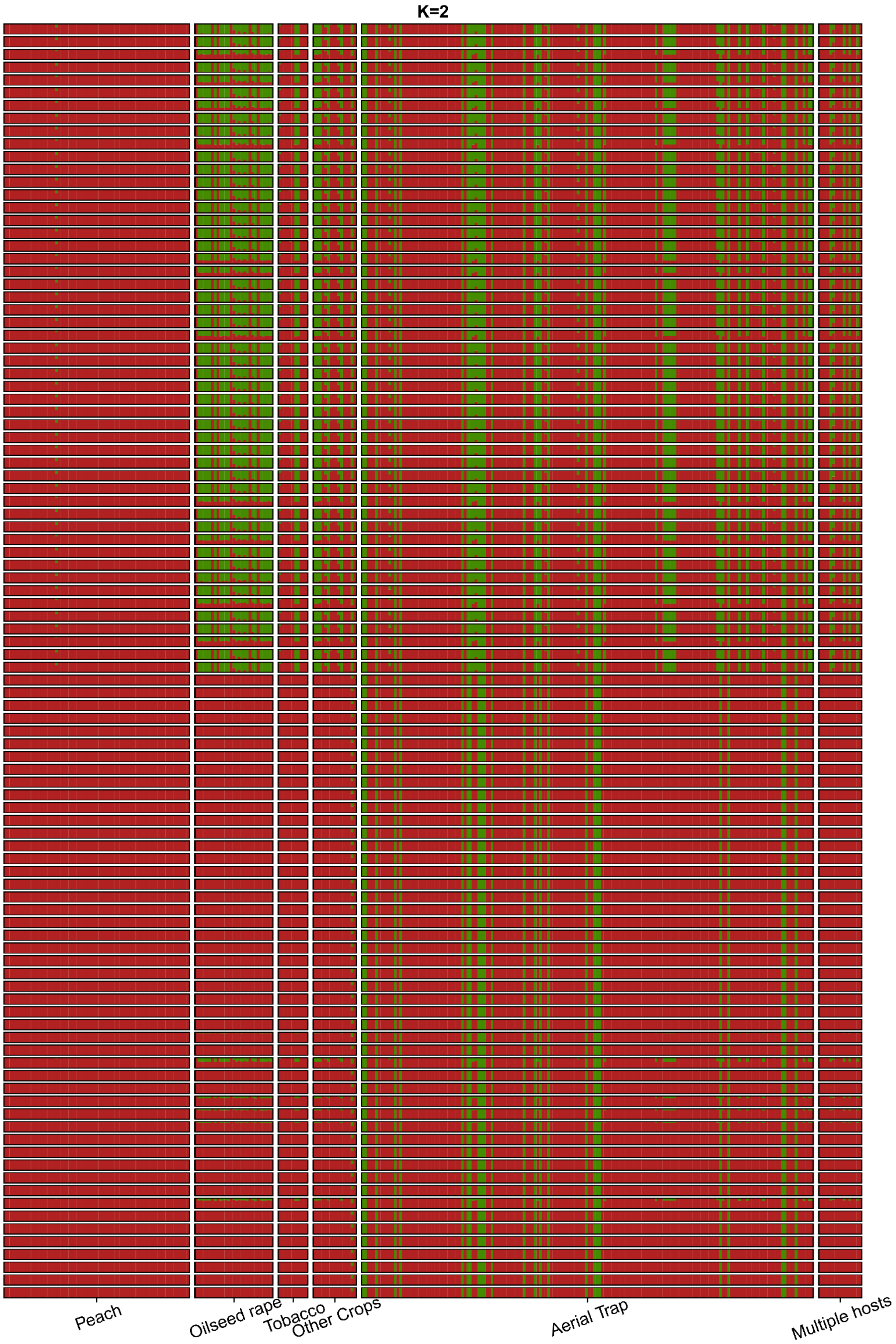
K=2
Peach
Oilseed rape
Tobacco
Other Crops
Aerial Trap
Multiple hosts

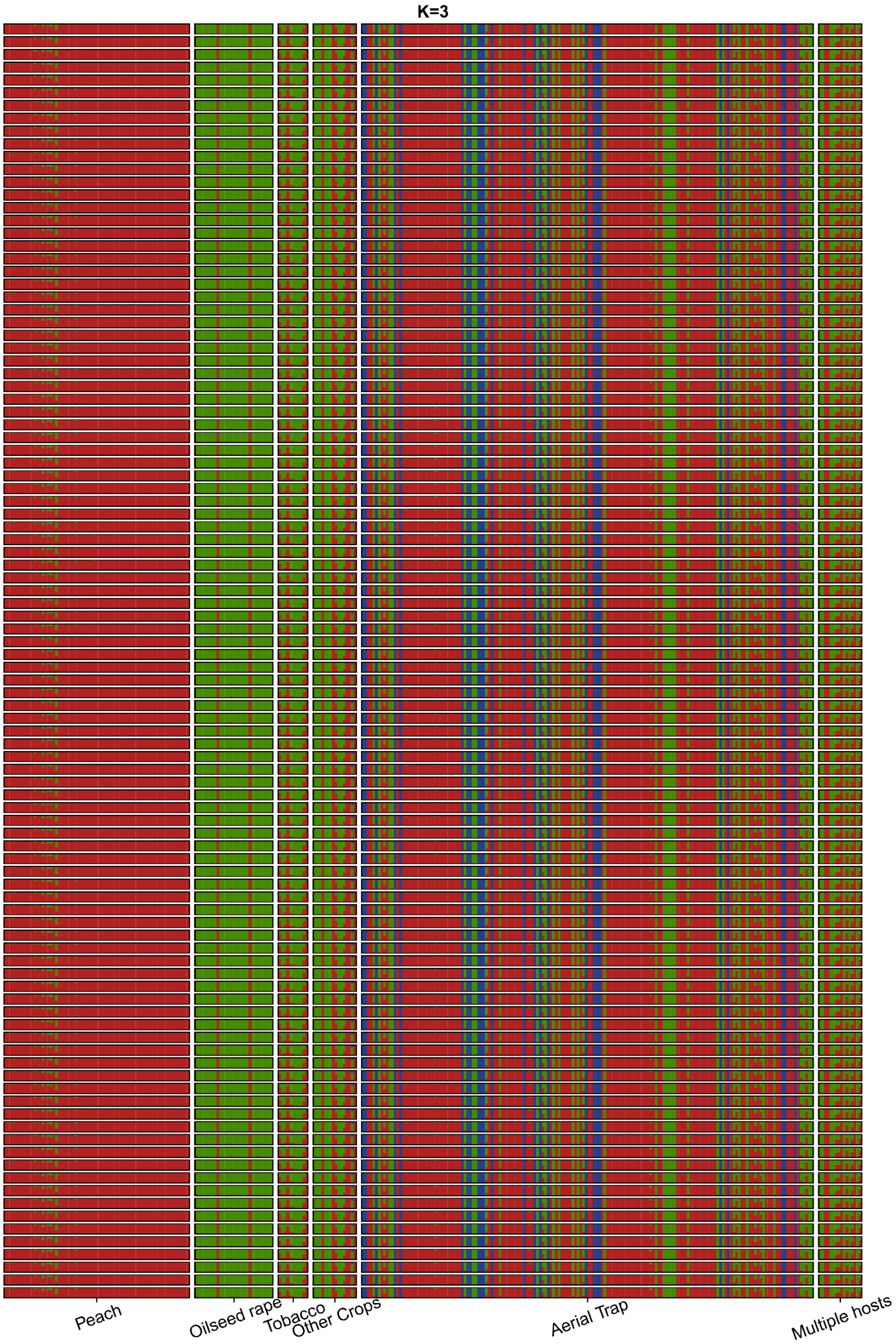
K=3
Peach
Oilseed rape
Tobacco
Other Crops
Aerial Trap
Multiple hosts

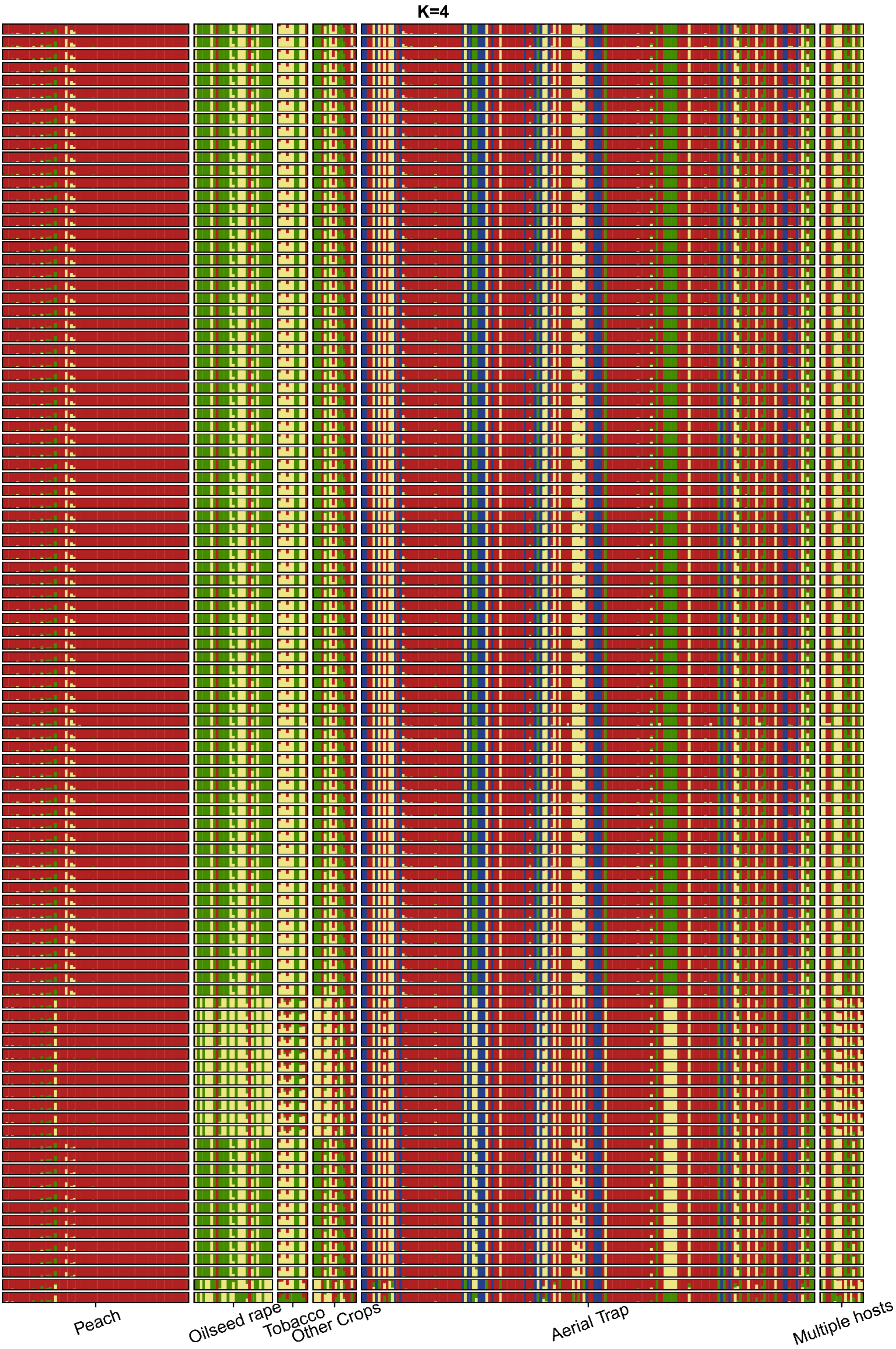
K=4
Peach
Oilseed rape
Tobacco
Other Crops
Aerial Trap
Multiple hosts

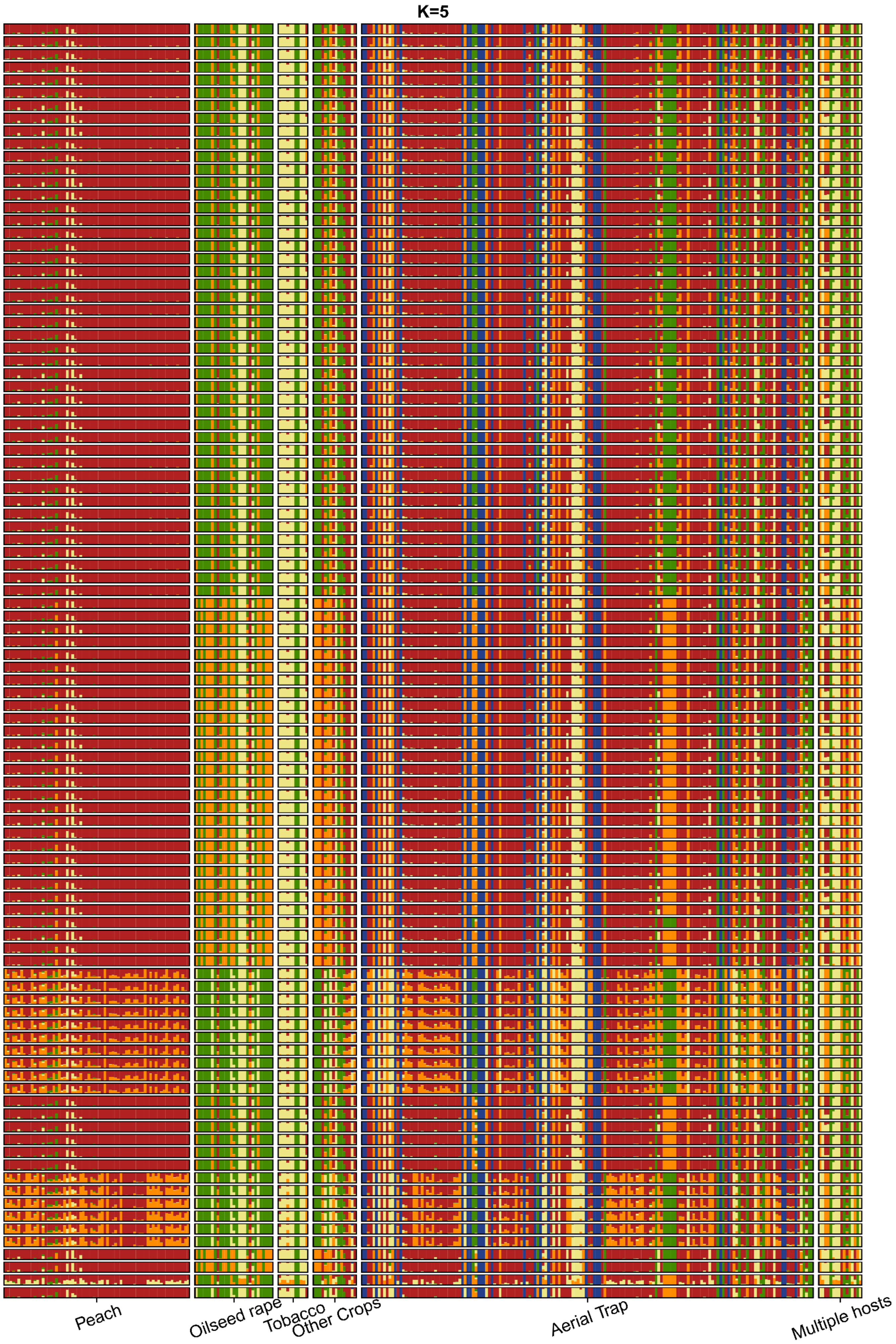
K=5
Peach
Oilseed rape
Tobacco
Other Crops
Aerial Trap
Multiple hosts

K=6

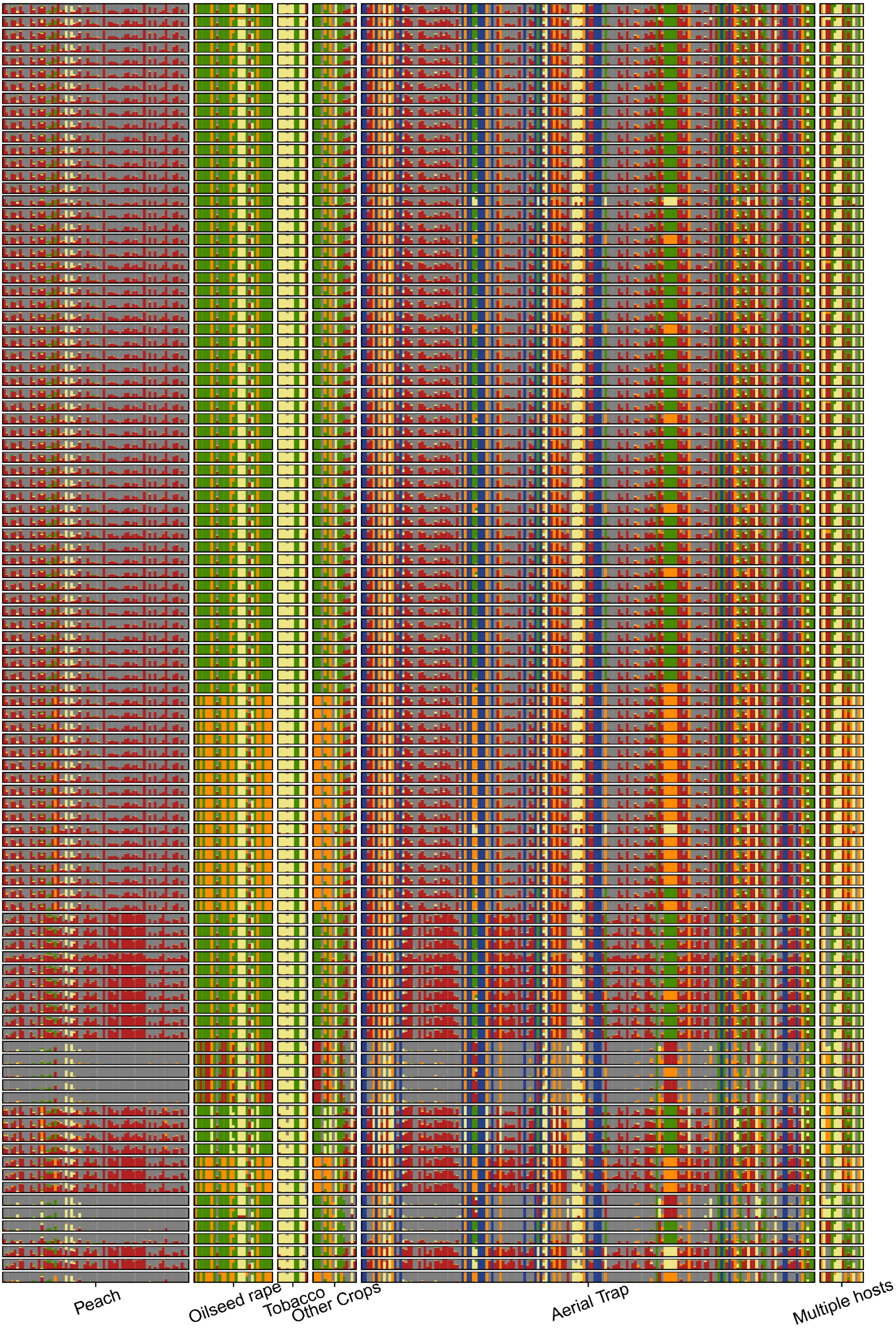
Peach
Oilseed rape
Tobacco
Other Crops
Aerial Trap
Multiple hosts

Figure S4: Q-plot for the 100 runs of the Bayesian clustering (STRUCTURE) analyses for K=2 to K=6.

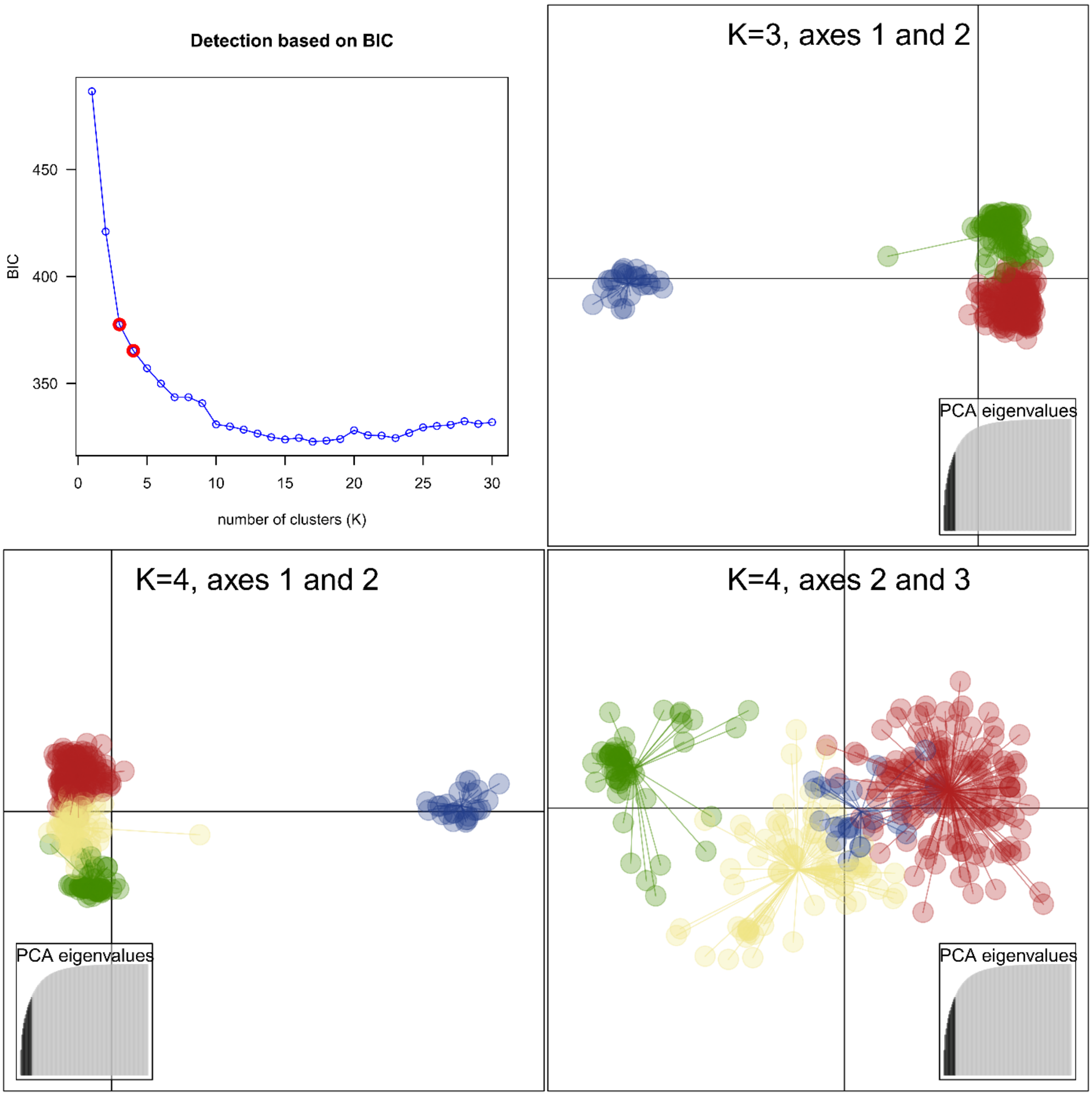

Figure S5: Scatter plot of the DAPC analyses for K=3 and K=4. The K value of interest were chosen based on BIC variations (emphasized on the graph with red dots). The scatter plot is displayed for axes 1 and 2 for K=3 and for axes 1 and 2 and 2 and 3 for K=4. The different colors represent the different genetic clusters as identified by DAPC.

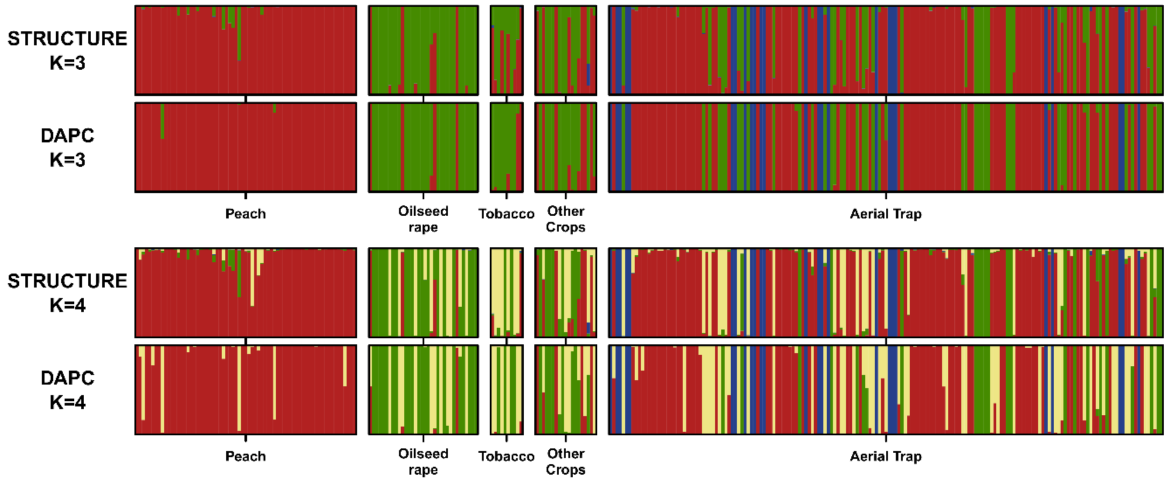

Figure S6: Comparison of the Bayesian clustering analysis and DAPC results for K=3 and K=4. The comparison is made on the clone-corrected dataset (n=305).

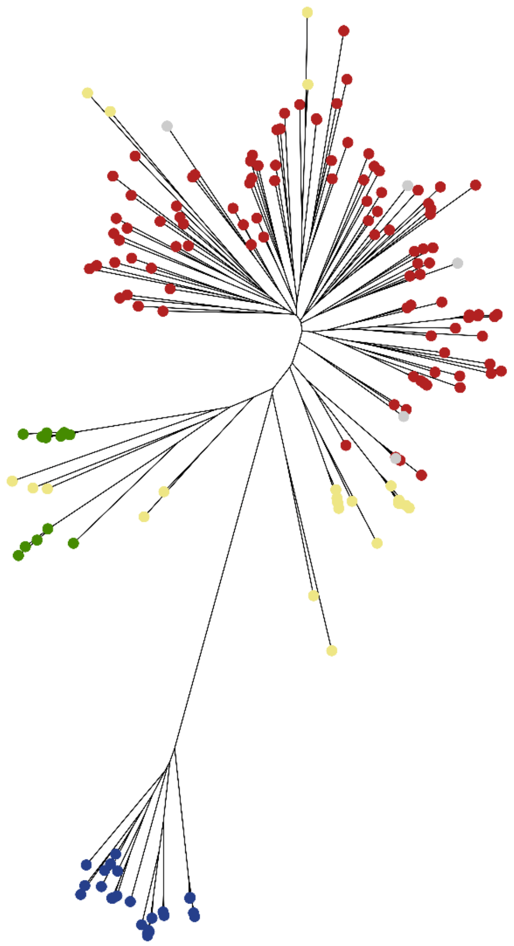

Figure S7: Neighbor joining tree based on shared allele distances between individual MLG sampled in the aerial trap. The colors of the tip represent the membership of the MLG to the corresponding genetic cluster.

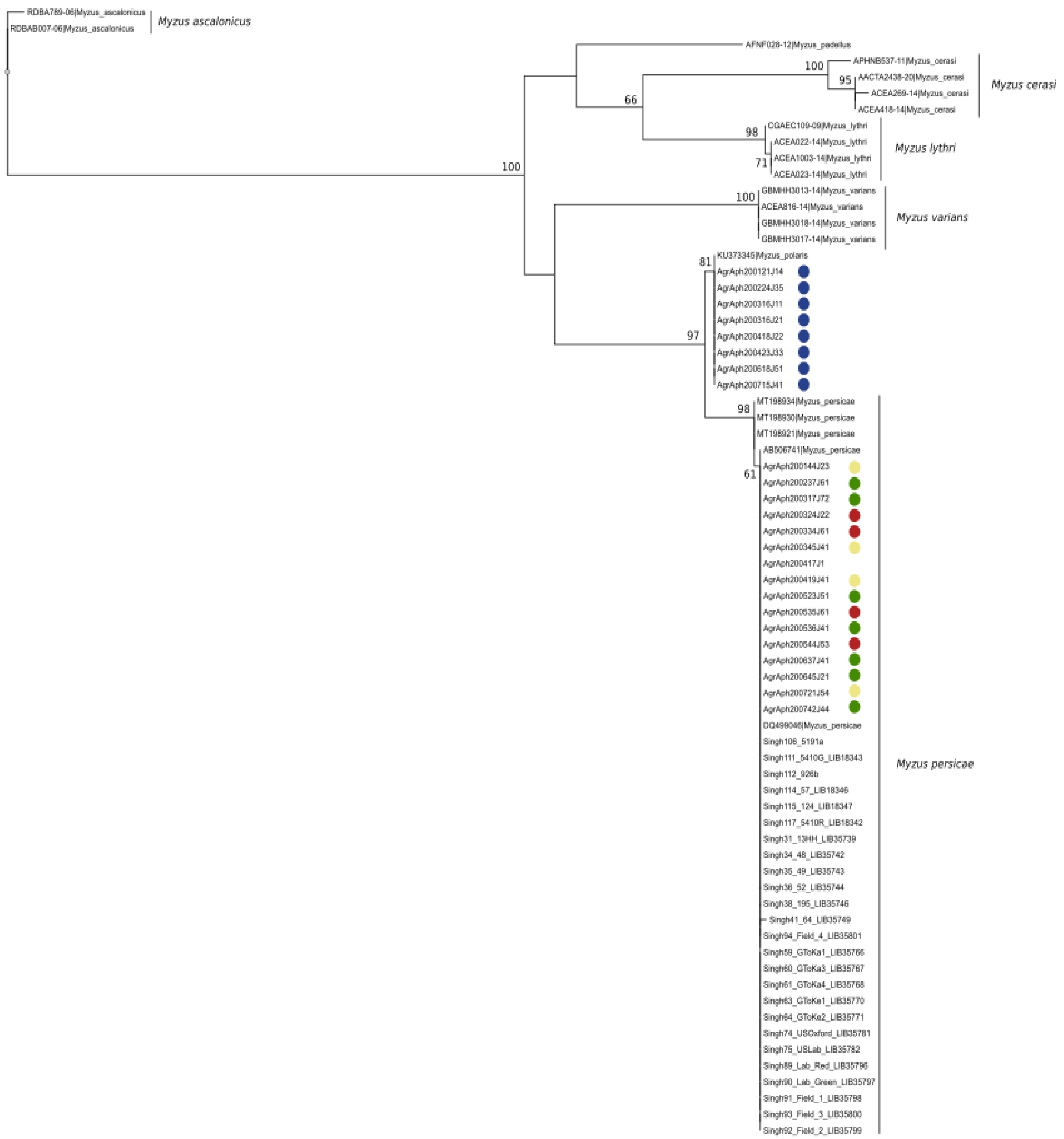

Figure S8: Phylogenetic topology obtained by the Maximum Likelihood (ML) method with the GTR+G model based on the alignment of CO1 sequences from a subsample of aphids collected by the aerial trap (individuals with only digits in the identifier). Model selection was performed based on the BIC criterion. The colored discs indicate the cluster assigned with q>0.7 to each individual in the aerial sample. Individuals with identifiers beginning with 'Singh' are clones that were collected from tobacco worldwide and form a separate clade in the phylogenetic analysis of Singh et al. (2021). The remaining individuals are from the Genbank or BOLD database and are labeled with an identifier

containing followed by their species name. The values at the nodes are percentages of 500 bootstraps.

Table S1: Pairwise Fst between genetic clusters. All pairwise genotypic differentiations were found to be highly significant (P<0.001).

	Red Cluster	Green Cluster	Yellow Cluster
Green Cluster	0.274	0	
Yellow Cluster	0.121	0.153	0
Blue Cluster	0.525	0.548	0.506

Table S2. Number of MLGs (individuals) carrying MACE and / or 918L in the different datasets. Here we consider both 918L alleles (L-ttg and L-ctg) and either zygosity status for each allele.

**Genotype**		**Air data**	**Peach**	**Tobacco**	**Oilseed rape**	**Other crops**
**MACE 431F**	**skr 918L**					
absent	absent	118 (346)	47 (64)	3 (24)	9 (98)	5 (5)
absent	present	1 (1)	19 (33)	0 (0)	2 (3)	6 (6)
present	absent	2 (5)	2 (2)	1 (1)	1 (1)	4 (6)
present	present	10 (54)	1 (3)	2 (21)	25 (500)	9 (59)

Table S3. Information on the 3-loci resistotypes found on crops. RG0 to RG10 were recorded from both the aerial and field samples. RG11 to RG15 were only recorded from crops. *kdr,* residue 1014 on VGSC, *skdr*, residue 918 on VGSC, MACE, residue 431 on AChE. Resistotypes highlighted in grey are dual-target mutants (they carry at least one mutation in the voltage-dependent sodium channel and one in acetylcholinesterase).

**Resisto-type ID**	**Nucleotidic genotypes (2 alleles per codon; *kdr* - *skdr* - MACE)**	**Resistance genotypes**			**Percentage of each resistotype recorded on crops**			
		kdr	***skdr***	**MACE**	**oilseed rape (NE France; 2009-2011)**	**other crops (Rhône Valley; 2014)**	**peach (Rhône Valley; 2013)**	**tobacco (France; 2003-2008)**
RG0	CTC/CTC-ATG/ATG-TCA/TCA	ss	ss	ss	0.3	1.3	0	41.3
RG1	TTC/CTC-ATG/ATG-TCA/TCA	sr	ss	ss	15.8	4	0	0
RG2	CTC/CTC-TTG/ATG-TCA/TTT	ss	sr (ML)	sr	82.3	78.7	0	45.7
RG3	TTC/CTC-ATG/ATG-TCA/TTT	sr	ss	sr	0	1.3	0	0
RG4	CTC/CTC-ATG/ATG-TCA/TTT	ss	ss	sr	0.2	0	0	2.2
RG5	CTC/CTC-CTG/ATG-TCA/TCA	ss	sr (ML)	ss	0	2.7	1	0
RG6	TTC/CTC-ACG/ATG-TCA/TCA	sr	sr (MT)	ss	0	0	16.7	0
RG7	TTC/TTC-ACG/ACG-TCA/TCA	rr	rr (TT)	ss	0	0	46.1	0
RG8	TTC/CTC-ATA/ATG-TCA/TCA	sr	s? (MI)	ss	0.9	0	0	10.9
RG9	TTC/CTC-ACG/CTG-TCA/TCA	sr	rr (LT)	ss	0	2.7	27.5	0
RG10	CTC/CTC-TTG/ATG-TCA/TCA	ss	sr (ML)	ss	0.3	0	0	0
RG11	CTC/CTT-ATG/ACG-TCA/TTT	sr	sr (MT)	sr	0	6.7	1	0
RG12	CTC/CTT-TTG/ACG-TCA/TCA	sr	rr (TL)	ss	0.2	1.3	3.9	0
RG13	CTC/CTC-CTG/CTG-TCA/TCA	ss	rr (L'L')	ss	0	1.3	0	0
RG14	CTT/CTT-ACG/ACG-TCA/TTT	rr	rr (TT)	sr	0	0	1	0
RG15	CTC/CTT-CTG/ACG-TCA/TTT	sr	rr (L'T)	sr	0	0	2.9	0

* ss. sensitive homozygous. sr. heterozygous. rr. resistant homozygous (as known from literature in terms of associated phenotype). ?? unknown resistance phenotype; kdr: s = L. r = F; s-kdr. s=M. r=T. L. I. Mace. s=S. r=F

** Exclusive of the genotyped individuals which did not allow to get the full 3-loci RG (11.2% aerial samples)

Supplementary bibliography

Jin, L. and Chakraborty, R., 1994. Estimation of genetic distance and coefficient of gene diversity from single-probe multilocus DNA fingerprinting data. *Molecular Biology and Evolution*, *11*(1), pp.120-127.

Kopelman, N.M., Mayzel, J., Jakobsson, M., Rosenberg, N.A. and Mayrose, I., 2015. Clumpak: a program for identifying clustering modes and packaging population structure inferences across K. *Molecular ecology resources*, *15*(5), pp.1179-1191.

Langella, O., 2007. Populations 1.2. 30: Population genetic software (individuals or populations distances, phylogenetic trees). *http://www. bioinformatics. org/~ tryphon/populations/*.

Paradis, E. and Schliep, K., 2019. ape 5.0: an environment for modern phylogenetics and evolutionary analyses in R. *Bioinformatics*, *35*(3), pp.526-528.
